# LncRNA Gas5, a Target of the Nonsense-Mediated Decay Pathway in the Brain, Regulates Neuroinflammation and Neurodegenerative Disease Pathways in a Tauopathy Mouse Model

**DOI:** 10.3390/ijms27177675

**Published:** 2026-08-27

**Authors:** Meredith Krause-Hauch, Bangmei Wang, Rekha S. Patel, Laura Verdina, Ashley Hedrick, Laura J. Blair, Ashutosh Dharap, Jianfeng Cai, Niketa A. Patel

**Affiliations:** 1 Research Service, James A. Haley Veterans’ Hospital, Tampa, FL 33612, USA; meredith.krause-hauch@va.gov (M.K.-H.); bangmei.wang@va.gov (B.W.); rekha.patel1@va.gov (R.S.P.); laura.blair@va.gov (L.J.B.); 2 Byrd Alzheimer’s Center and Research Institute, Tampa, FL 33613, USA; lauraverdina@usf.edu (L.V.); ahedrick1@usf.edu (A.H.); dharap@usf.edu (A.D.); 3 Department of Molecular Medicine, Morsani College of Medicine, University of South Florida, Tampa, FL 33612, USA; 4 Department of Chemistry, University of South Florida, Tampa, FL 33612, USA; jianfengcai@usf.edu

**Keywords:** nonsense-mediated decay, GAS5, lncRNAs, tauopathy, neuroinflammation, digital spatial transcriptomics

## Abstract

Dysregulation of target genes of nonsense-mediated decay (NMD) in the brain remains sparsely known in tauopathies. PS19 transgenic mice expressing human mutant P301S tau were evaluated for levels of lncRNA Gas5, a target of NMD. The results show Gas5 decreased in the brains of PS19 mice as they aged. We evaluated the consequences of blocking the NMD-mediated turnover of Gas5 using a small molecule administered intranasally to PS19 mice. The results show NPC86 disassociated Upf1 and Gas5, thereby hindering NMD. NPC86 treatment increased Gas5 levels in the cortex of male PS19 mice concurrent with a highly significant decrease in pTau S214 and neuroinflammatory genes while increasing insulin signaling. Consequently, digital spatial profiling identified Gas5-regulated genes and pathways. NPC86 treatment enhanced neuronal homeostasis, synaptic vesicle transport and mitochondrial function and downregulated neuroinflammatory pathways. The nodal genes in Parkinson’s signaling pathway, the multiple sclerosis signaling pathway, the Gα(s) signaling pathway, the neuroinflammation signaling pathway, and the interferon gamma signaling pathway were decreased in response to NPC86. The study demonstrates the potential of selectively stabilizing NMD-target lncRNA Gas5 levels to alleviate early neurodegenerative pathology in tauopathy.

## 1. Introduction

Pathological accumulation of tau in the brain is a hallmark of tauopathies such as progressive supranuclear palsy (PSP), chronic traumatic encephalopathy (CTE), Parkinson’s disease (PD), frontotemporal degeneration (FTD), frontotemporal dementia and parkinsonism linked to chromosome 17 (FTDP-17). The FTD group of disorders affects people younger than 60 years, with 15–20 affected individuals per 100,000 in the US. However, the economic burden of FTD is higher than the per-patient costs of AD [1]. Mutations, such as frontotemporal dementia-associated P301S, promote abnormal hyperphosphorylation and the aggregation of tau. PS19 mice are tau transgenic mice that express human mutant P301S tau at a five-fold higher level than endogenous mouse tau.

The NMD pathway is critical for identifying and targeting transcripts for turnover, thereby performing a crucial surveillance mechanism to degrade transcripts that are mis-spliced or have genetic mutations. Importantly, almost 10% of normal transcripts are regulated by NMD as part of the physiological regulation of lncRNAs, miRNA and snoRNA [2]. NMD complexes comprise the core proteins Upf1, Upf2, and Upf3, which interact with the eukaryotic release factors eRF1 and eRF3, and exon junction complex proteins eIF4A3, RBM8A, MAGOH and MLN51. Upf1 is a central initiator protein in the NMD pathway [3]. NMD regulates neuronal development [4] as well as axonal migration [5] and synaptic protein levels [6,7]. Mutations in neurofibrillary filaments and tau marks them for NMD [8,9].

The lncRNA growth-arrest specific transcript 5 (Gas5) regulates insulin signaling in the normal brain [10]. The levels of Gas5 are often determined by its rate of mRNA turnover, which is governed by the NMD pathway [11]. Using BrU pulse-chase labeling [12], it was demonstrated that Gas5 levels are regulated by NMD. Our results shown in this manuscript demonstrate that Gas5 levels are lower in PS19 mice than in non-transgenic littermates. Inhibiting the core NMD mechanism or depletion of Upf1 is not recommended due to its central role in the post-transcriptional regulation of genes and as a crucial surveillance mechanism. We previously developed a Gas5-stabilizing small molecule, NPC86, which blocks the binding of Upf1 to the 3′ region of Gas5, to specifically hinder NMD-mediated Gas5 turnover [13]. Furthermore, our results showed that intranasal delivery of NPC86 crosses the blood–brain barrier and is distributed across the brain regions, is not toxic, is highly specific for Gas5 and increased the levels of Gas5 in the brain [10]. Hence, we utilized NPC86 as a molecular tool to evaluate the consequences of blocking NMD-mediated turnover of Gas5 in the PS19 tau transgenic mouse model.

## 2. Results

### 2.1. Gas5 Levels Are Lower in the Brain of PS19 Mice

**Figure 1:**
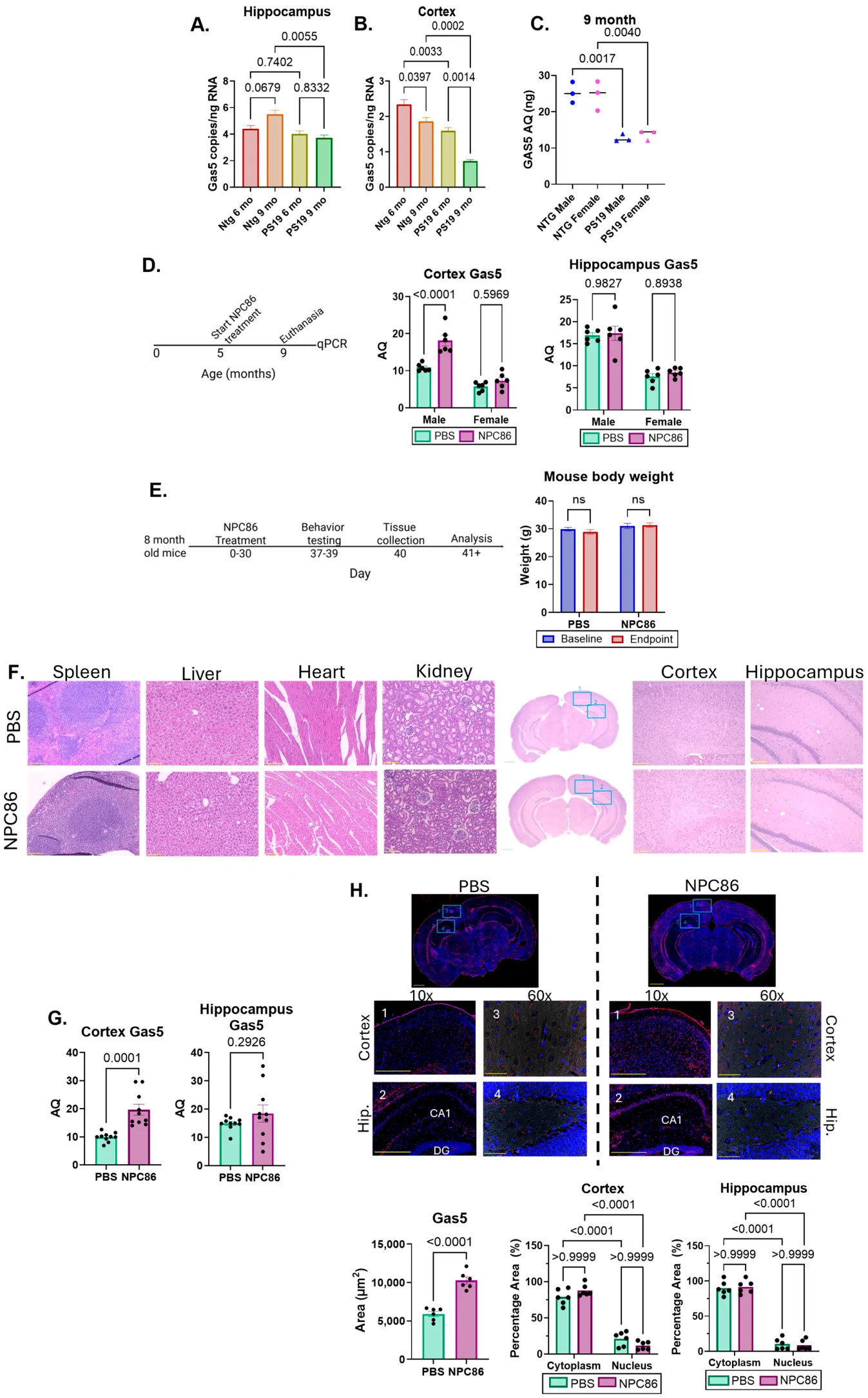
ddPCR was performed to quantify Gas5 in the brains of non-transgenic (Ntg) and PS19 mice at 6 and 9 months of age in the (**A**) hippocampus and (**B**) cortex. The results are in copies of Gas5/ng RNA. The number of Gas5 copies was determined by the copies present in each droplet. Gas5 copies in 404–1350 droplets were quantified. The copies per well were divided by the amount of RNA used, and copies per ng RNA was calculated. *n* = 6 mice. Analysis completed using one-way ANOVA. Red = Ntg 6 mo; orange = Ntg 9 mo; yellow = PS19 6 mo, green = PS19 9 mo. (**C**) RT-qPCR was performed to compare the absolute quantification (AQ) of Gas5 in the cortex of 9-month-old male and female Ntg and PS19 mice. *n* = 3. Statistical analysis was performed using one-way ANOVA. Blue points are male, and pink points are female; circles are Ntg, and triangles are PS19. (**D**) Five-month-old male and female PS19 mice were administered NPC86 or PBS (vehicle) intranasally once weekly for 4 months. RT-qPCR was performed using Gas5 primers, and the absolute quantification (AQ in ng) was calculated for the cortex and hippocampus. Cortex samples were analyzed by sex. *n* = 6. Statistical analysis was performed using two-way ANOVA. Green bars are Ntg, and purple bars are NPC86. (**E**) Eight-month-old PS19 mice treated intranasally with either PBS or NPC86 for 1 month. The body weight for PBS- and NPC86-treated mice at baseline and at experimental endpoint was evaluated. *n* = 12. Statistical analysis was performed using two-way ANOVA. Blue bars are baseline weight measurements, and red bars are endpoint weight measurements. ns = not significant. (**F**) Representative images of H&E staining of the spleen, liver, heart, kidneys (20× magnification, scale bar = 100 µm) and brain (10× magnification, scale bar = 1 mm), with insets showing the cortex and hippocampus (10× magnification, scale bar = 200 µm). In the whole brain images, the numbered boxes correspond to the brain region: 1 corresponds to the cortex, and 2 corresponds to the hippocampus. (**G**) RT-qPCR was performed to compare the AQ of Gas5 in the cortex and hippocampus of 9-month-old male PS19 mice after 1-month intranasal treatment with either PBS or NPC86. *n* = 10. Analysis completed using Student’s unpaired t-test. Green bars are Ntg, and purple bars are NPC86. (**H**) RNAscope was performed to visualize Gas5 abundance in the brain of PS19 with PBS or NPC86 treatment. Blue = DAPI, Pink = Gas5. Images are taken on the Keyence BZ810 at 4× (scale bar = 1 mm), 10× (scale bar = 500 µm) and 60× magnification (scale bar = 50 µm), and representative images are shown, with CA1 and dentate gyrus (DG) regions of the hippocampus labeled. In the 10× images, the numbered boxes correspond to the brain region in the 20× images: 1 and 3 corresponds to the cortex, and 2 and 4 corresponds to the hippocampus. Quantification of Gas5 levels was performed on 12 equally sized fields uniformly distributed across tissue sections for each treatment, and the average was calculated for each mouse. Analysis was performed using Keyence Software (v1.1.1.8). Results are shown as the area (µm^2^) of Gas5 fluorescence. Statistical analysis performed using Student’s t-test. The percent area of Gas5 within the cytoplasm and nucleus of the cortex and hippocampus with 60× magnification was quantified using Keyence Software. *n* = 6 mice. Analysis completed using two-way ANOVA. Green bars are Ntg, and purple bars are NPC86.

We evaluated the levels of Gas5 in PS19 mice compared to the non-transgenic (Ntg) age-matched littermates at ages 6 months and 9 months. The results demonstrate that PS19 mice have significantly decreased Gas5 levels at 9 months in both the hippocampus (Figure 1A) and cortex compared to Ntg. Comparing the levels of Gas5 between ages 6 and 9 months of the PS19 mice, the results from the hippocampus did not show a significant decrease at 9 months compared to Gas5 levels at 6 months in the PS19 mice. However, the cortex had significantly lower levels at 9 months compared to 6 months (Figure 1B). Both male and female PS19 mice had lower Gas5 levels in the cortex than Ntg (Figure 1C).

### 2.2. Intranasal Administration of NPC86 Increases Gas5 Levels in PS19 Mice

Previously, we showed [10] that NPC86 administered intranasally distributes uniformly across the brain regions and increases the levels of Gas5 in a dose-dependent manner. Thus, in the group 1 cohort, we treated PS19 mice with intranasal NPC86 starting at 5 months and evaluated the levels of Gas5 at 9 months. The response to NPC86 was not significant in either male or female mice in the hippocampus. NPC86 increased Gas5 levels in the cortex in male PS19 during aging from 5 months to 9 months, while female mice did not show a significant response (Figure 1D). Based on this observation, we focused our group 2 study on male PS19 mice.

Next, in our group 2 cohort, to evaluate the effect of stabilizing Gas5 levels when tau pathology is emerging [14,15,16,17], we administered NPC86 intranasally every 7 days for 4 weeks to male PS19 mice at 8 months of age. NPC86 treatment did not affect the body weight and showed no toxicity in the brain or other organs (Figure 1E,F). The results demonstrate significantly higher levels of Gas5 in response to NPC86 (Figure 1G) in the cortex, while the response was not statistically significant in the hippocampus. RNAscope results also demonstrate an increase in Gas5 levels in response to NPC86 (Figure 1H). Furthermore, the results demonstrate higher cytoplasmic levels of Gas5 in the cortex and hippocampus than in the nucleus. From here onwards, we evaluated the cortex of 9-month-old male PS19 mice with intranasal NPC86 administration. All data presented from this point were generated from the group 2 cohort.

### 2.3. NPC86 Blocks NMD-Mediated Turnover of Gas5

**Figure 2:**
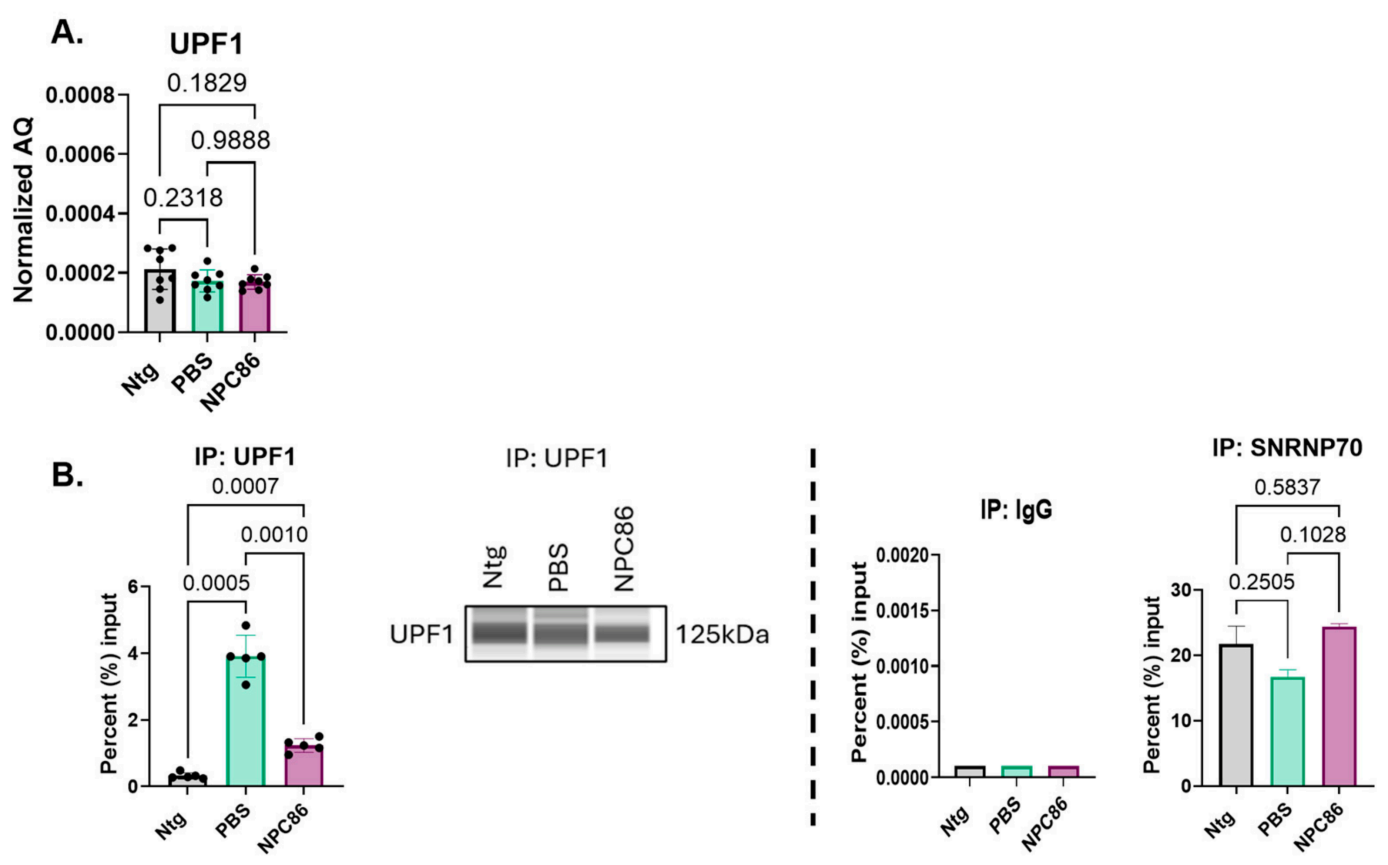
(**A**) UPF1 levels were evaluated in the cortices of Ntg and PS19 mice treated with NPC86 or PBS using SYBR Green RT-qPCR. AQ = absolute quantification in ng. *n* = 8. Statistical analysis using one-way ANOVA. (**B**) RNA-immunoprecipitation (RIP) assay was performed for cortex samples of Ntg or PS19 mice treated with NPC86 or PBS using Upf1 antibody for immunoprecipitation. Equal Upf1 immunoprecipitation was evaluated using the ProteinSimple Jess Automated Western Blot System (BioTechne, Minneapolis, MN, USA). The image shows the pseudo-blot (lane view) image generated for the Upf1 antibody and is representative of five samples per group. qPCR was performed using Gas5 primers (*n* = 5). Simultaneously, SNRNP70 (positive control) was immunoprecipitated and qPCR performed using U1 primers or IgG (negative control) was immunoprecipitated followed by qPCR using Gas5 primers. The graph represents percent input calculated using the formula %input = 100 × 2^(Input Ct−IP Ct)^, where Input Ct is the total Gas5 Ct value in the sample before the immunoprecipitation, and IP Ct is the Gas5 Ct value after the RIP assay. Statistical analysis using one-way ANOVA. In all panels, gray bars represent Ntg, green bars represent PBS and purple bars represent NPC86.

We evaluated the levels of Upf1, the initiator of the NMD pathway, and the results (Figure 2A) demonstrate no significant change in Upf1 levels in the cortex when comparing Ntg and PS19 with PBS (vehicle) or NPC86 treatment. Next, we performed an RNA-immunoprecipitation (RIP) assay to evaluate the association between Gas5 and Upf1 in the cortex. Our results (Figure 2B) demonstrate an increased association of Upf1 with Gas5 in PS19 compared to Ntg. NPC86 treatment blocks the association of Upf1 with Gas5, as shown in the RIP assay. The full UPF1 blot can be found in Supplemental Figure S1.

### 2.4. NPC86 Treatment Alleviates Neuronal Insulin Signaling and Decreases Tau Phosphorylation

**Figure 3:**
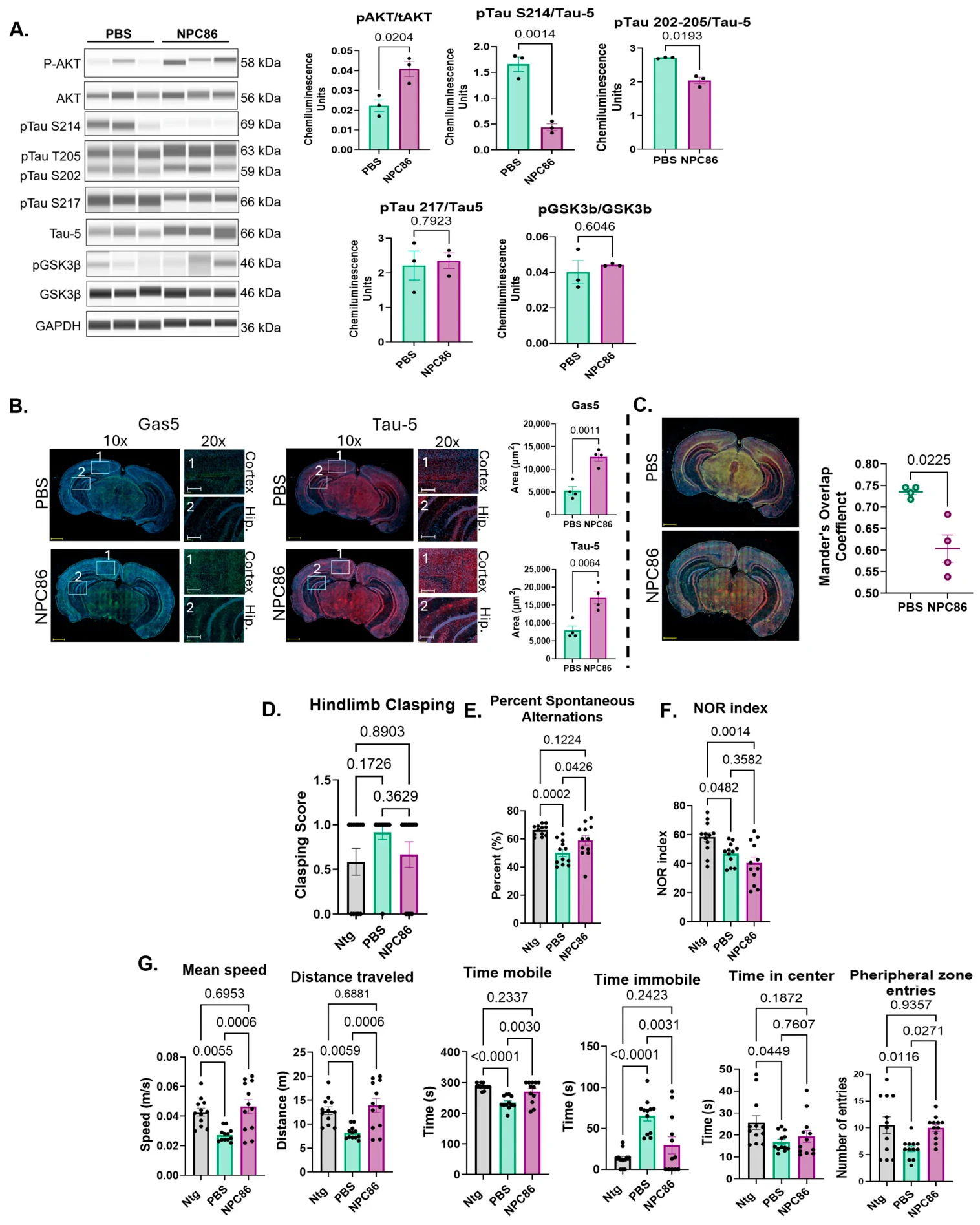
(**A**) Immunoblotting of the cortex samples from PS19 mice treated with PBS or NPC86 were performed using the ProteinSimple Jess Automated Western Blot System (BioTechne, Minneapolis, MN, USA) and analyzed with its built-in software, Compass for Simple Western (v. 7.0). The image shows the pseudo-blot (lane view) image generated for each antibody (as indicated in the figure) by Compass software from the capillary signals. Jess measures total protein in every capillary and normalizes the signal against actual protein load, reported as chemiluminescent units. Next, each antibody’s chemiluminescent units were normalized to the Gapdh chemiluminescent units and plotted on the graph. *n* = 3. Statistical analysis was performed using one-way ANOVA. (**B**) RNA-FISH/IF was performed with DAPI counterstain (blue) to visualize Gas5 (green) and Tau5 (red) abundance in the brain; images taken at 10× (scale bar = 1 mm) and 20× (scale bar = 200 µm). In the 10× images, the numbered boxes correspond to the brain region in the 20× images: 1 corresponds to the cortex, and 2 corresponds to the hippocampus. The graph shows quantification of Gas5 abundance. Four tissue samples per group were analyzed. The average area of Gas5 abundance was quantified from 12 equally sized fields, which were equally distributed across each tissue section. *n* = 4 mice. Statistical analysis using Student’s t-test. (**C**) Images were overlayed to visualize areas of colocalization of Gas5 (green) and Tau5 (red) in the brain, as indicated by yellow; images taken at 10× (scale bar = 1 mm). Mander’s overlap coefficient was calculated to evaluate the colocalization between Gas5 and Tau-5. *n* = 4. Analyzed by Student’s unpaired t-test. (**D**) One week after the final NPC86 treatment, behavioral testing was completed to evaluate memory and motor abilities. Hindlimb clasping was completed by suspending mice by the tail for 5 s to evaluate limb placement. Each mouse was scored 0–3, with 0 indicating normal function and 3 indicating severe dysfunction. PS19 mice were compared to non-transgenic (Ntg) littermates to assess changes in PS19 mice with and without NPC86 treatment. (**E**) Y-maze testing was completed and the number of times a mouse entered the three arms of the Y-maze without returning to a previously entered arm (spontaneous alternations) was determined and the percentage of spontaneous alternations was calculated. (**F**) Mice underwent novel object recognition (NOR) testing to evaluate memory over 3 days (habituation, training, testing). The NOR index was calculated as [(time spent exploring novel object) − (time spent exploring familiar object)]/(total exploration time). (**G**) To assess mobility and locomotor function, open field testing was completed. Mice were allowed to explore the testing arena for 15 min while recorded. Data was analyzed to evaluate distance traveled, mean speed, time mobile, time immobile, time spent in the center of the arena, number of peripheral zone (edge of arena) entries, and number of central zone (middle of arena) entries. *n* = 12. Statistical analysis using one-way ANOVA. In all panels, green bars represent PBS and purple bars represent NPC86. In panels (**D**–**G**), gray bars represent Ntg.

We previously demonstrated that Gas5 regulates insulin signaling in normal mice [10]. The results using the Jess Automated Western Blot System (Figure 3A; full blots can be found in Supplemental Figure S2) show that treatment with NPC86 increased the phosphorylation of Akt, thereby demonstrating increased insulin signaling in PS19 mice. Hyperphosphorylation of tau is an early post-translational modification, which promotes degeneration in tauopathies. The results show a highly significant decrease in pTauS214 with NPC86 treatment along with a significant decrease in pTauS202/T205. NPC86 did not change the phosphorylation of tau at Thr231, Ser404 or Ser416 (Supplemental Figure S3). The results also showed that pGSK3β, a prominent kinase phosphorylating tau, did not change significantly in response to NPC86 treatment. We performed RNA-FISH/IF to visualize localization and levels of Gas5 and tau in the brain. The results (Figure 3B) show that Gas5 (green; left panel) and Tau-5 (red; right panel) increased with NPC86 treatment. Next, analysis of the Mander’s overlap coefficient shows that colocalization of Gas5 and Tau-5 decreases with NPC86 treatment (Figure 3C).

As a test of motor function, hindlimb clasping was evaluated (0 = normal, 3 = dysfunction). The results show (Figure 3D) the score was slightly improved with NPC86 treatment, though not significantly. Next, the Y-maze test was used to evaluate short-term memory, and the results show (Figure 3E) a decreased percentage of spontaneous alternations in PS19 mice compared to age-matched Ntg mice. NPC86 treatment increased the percentage of spontaneous alternations in PS19 mice. In the novel object recognition (NOR) test, the results (Figure 3F) show high variability within the mice cohorts, and the difference between NPC86 treated and untreated PS19 mice was not statistically significant. Finally, the cohorts were evaluated for locomotion and emotionality using the ANY-maze open field test. The results (Figure 3G) show that NPC86-treated PS19 mice traveled a farther distance, had increased speed and explored the center more; they also had decreased time in the thigmotaxis region compared to the untreated PS19 mice.

### 2.5. Digital Spatial Profiling (DSP) of NPC86-Treated PS19 Mice

**Figure 4:**
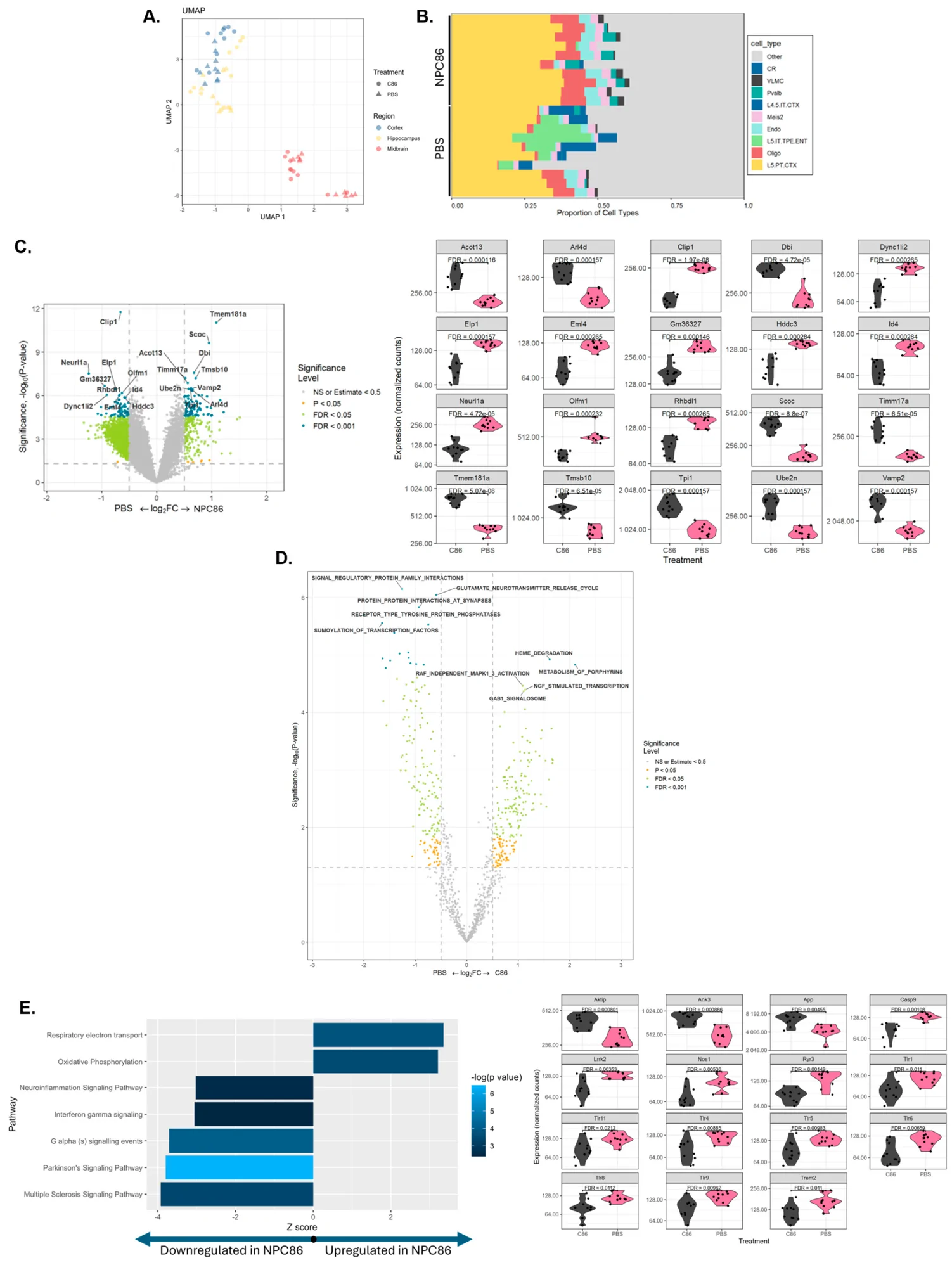
Digital spatial transcriptomics (DSP) of PS19 mice treated with PBS (Control) and NPC86. (**A**) UMAP visualizing the differences between PBS and NPC86 in the three sampled regions of the brain (cortex, hippocampus, midbrain). (**B**) Proportions of cell types present in the cortexes of PBS vs. NPC86 treated mice. (**C**) Top significant differentially expressed genes (DEGs) visualized by volcano plot and violin plots. In the volcano plot, points are graphed as −log(*p*-value) by log2 fold change (FC). DEGs were considered significant with *p*-value < 0.05 and |log2FC| > 0.5. Positive log2FC indicates genes are upregulated in NPC86 compared to PBS, while negative log2FC indicates DEGs are downregulated in NPC86 compared to PBS. In the violin plots, Gray = NPC86, Pink = PBS. Results are shown as normalized counts, and significance is shown as false discovery rate (FDR). (**D**) Top significant pathways (FDR < 0.001) were determined via single-sample gene set enrichment analysis (ssGSEA). Volcano plot of top pathways. Points are graphed as −log(*p*-value) by log2 fold change (FC). Pathways were considered significant with *p*-value < 0.05 and |log2FC| > 0.5. Positive log2FC indicates pathways are upregulated in NPC86 compared to PBS, while negative log2FC indicates pathways are downregulated in NPC86 compared to PBS. (**E**) Overrepresentation analysis (ORA) pathways are generated using Qiagen Ingenuity Pathway Analysis (IPA) software (v. 01-22-01). Positive z-scores indicate pathways are upregulated in NPC86 compared to PBS, while negative z-scores indicate pathways are downregulated in NPC86 compared to PBS. The color of each bar corresponds to the −log(*p*-value). Figure created using the R ggplot2 package. Expression of selected genes of interest that occur in these pathways are shown in violin plots. Gray = NPC86, Pink = PBS. Results are shown as normalized counts, and significance is shown as false discovery rate (FDR).

GeoMx DSP was performed on three brain regions (cortex, hippocampus, and midbrain) to investigate the spatially resolved profiling of proteins in PS19 mice with NPC86 treatment or PBS (vehicle). A Uniform Manifold Approximation and Projection (UMAP) indicated clustering of the three brain regions (Figure 4A). In particular, samples from the midbrain are clustered further from those from the cortex and hippocampus. Samples from the cortex and hippocampus are clustered closely, indicating high similarity. Furthermore, there is separation between samples from mice treated with PBS and those treated with NPC86, particularly in the cortex. Due to samples from the cortex having a stronger response in terms of Gas5 levels to NPC86 than the hippocampus and midbrain, we focused the analysis on differentially expressed genes (DEGs) and their pathways in the cortex.

We compared the composition of cell types in the cortex of mice treated with PBS vs. NPC86 (Figure 4B). Analysis showed that there was a greater proportion of Layer 5 intratelencephalic glutamatergic neurons of the temporal processing entity in the entorhinal cortex (L5 IT TPE ENT) and Layer 4/5 intratelencephalic cortical cells (L4 5 IT CTX) in the PBS-treated cortex than in those treated with NPC86. However, there was a greater proportion of oligodendrocytes (Oligo), vascular leptomeningeal cells (VLMCs), and parvalbumin cells (Pvalb) in the NPC86-treated cortex region than in the PBS-treated region.

DSP analysis identified a total of 10,994 genes expressed in the cortex samples. Of these genes, 6462 genes significantly changed in the cortex of PS19 mice in response to NPC86 treatment at (False Discovery Rate) FDR < 0.05. Moreover, 1995 genes were significant using the dual thresholding of FDR < 0.05 and |log2 fold change| > 0.5. Differentially expressed gene (DEG) analysis revealed 168 highly differentially expressed genes with FDR < 0.001 (Figure 4C). Seventy genes were downregulated, while 98 genes were upregulated in response to NPC86 treatment. The top 10 DEGs (as determined by log2FC and FDR values) upregulated in response to NPC86 treatment compared to PBS were Tmem181a, Scoc, Dbi, Tmsb10, Vamp2, Acot13, Timm17a, Ube2n, Arl4d, and Tpi1, and the top 10 DEGs downregulated in response to NPC86 treatment compared to PBS were Clip1, Neurl1a, Elp1, Gm36327, Rhbdl1, Olfm1, Dync1li2, Eml4, Id4, and Hddc3.

We next performed ssGSEA for analysis of enriched pathways (Figure 4D). Amongst the pathways with an FDR < 0.001, the top five pathways upregulated in the cortex of PS19 mice treated with NPC86 compared to those treated with PBS included (1) heme degradation (5 DEGs), (2) metabolism of porphyrins (14 DEGs), (3) RAF-independent MAK1 3 activation (12 DEGs), (4) NGF-stimulated transcription (5 DEGs) and (5) GAB1 signalosome (11 DEGs). The top five pathways downregulated in the cortex of PS19 mice treated with NPC86 compared to PBS-treated PS19 mice are (1) signal regulatory protein family interactions (6 DEGs), (2) the glutamate neurotransmitter release cycle (21 DEGs), (3) protein–protein interactions at synapses (63 DEGs), (4) receptor-type tyrosine protein phosphatases (19 DEGs), and (5) sumoylation of transcription factors (11 DEGs). The DEGs found in each of the top pathways are listed in Table S4.

We then utilized Qiagen IPA (v.01-22-01; QIAGEN Inc., https://digitalinsights.qiagen.com/IPA, accessed 16 December 2025) [18] to investigate additional pathways via overrepresentation analysis (ORA). IPA pathway analysis revealed 225 significantly expressed pathways (−log(*p*-value) > 1.30). Of these pathways, 156 have a negative z-score, indicating that these pathways are downregulated in the cortex of mice treated with NPC86 compared to those treated with PBS. Figure 4E visualizes the significant IPA pathways as determined by the greatest |z-score| with *p*-value > 0.05. Of interest, specific neurodegenerative disease pathways that were downregulated with NPC86 treatment (negative z-score) included Parkinson’s signaling pathway, the neuroinflammation signaling pathway, the multiple sclerosis signaling pathway, the Gα(s) signaling pathway, and the interferon gamma signaling pathway.

**Figure 5:**
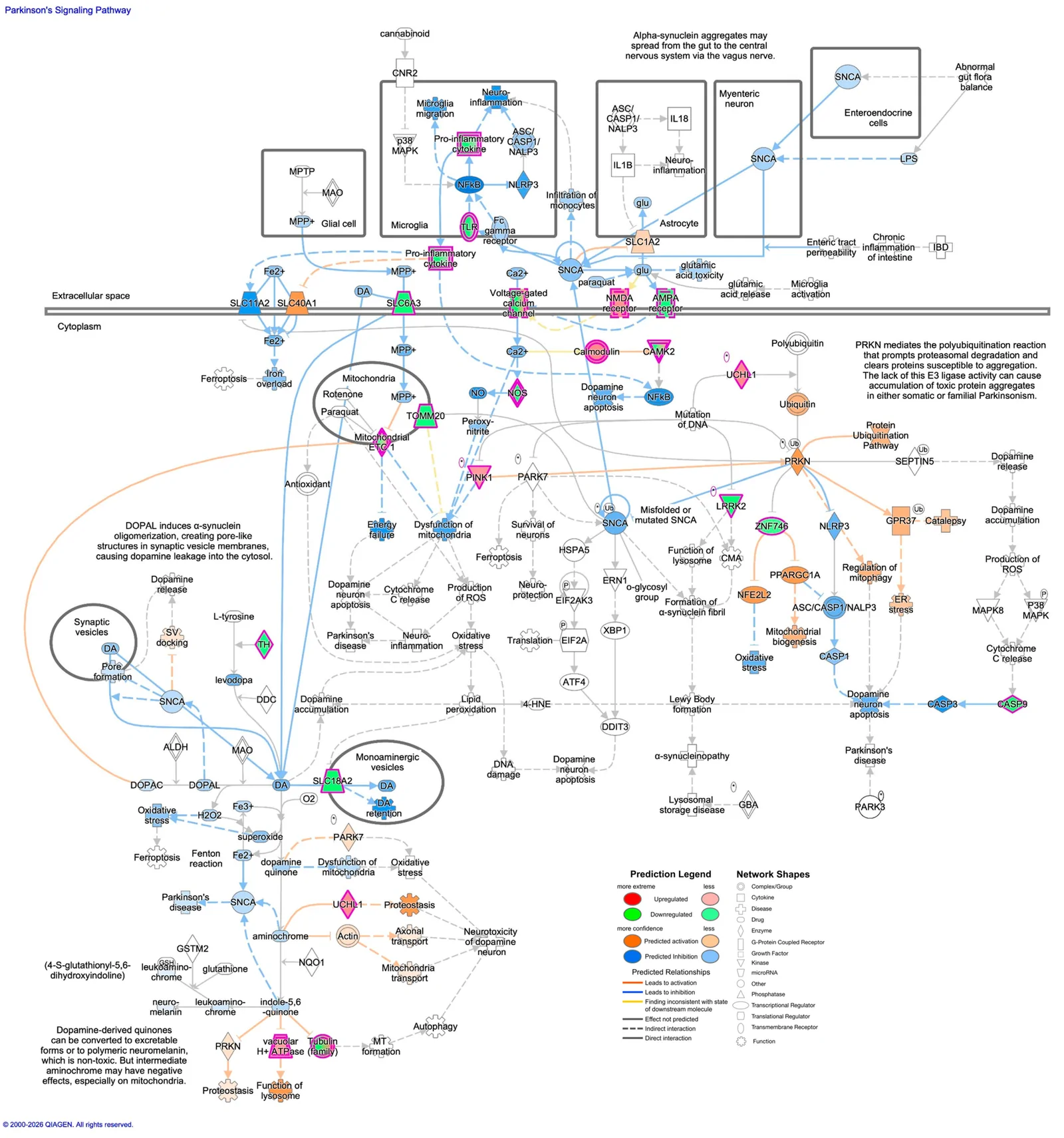
Parkinson’s disease pathway generated using Qiagen Ingenuity Pathway Analysis software v.01-22-01 (QIAGEN Inc., Germantown, MD, USA, https://digitalinsights.qiagen.com/IPA, accessed 16 December 2025). The asterisks (*) inside the oval indicates a node with strong disease-relevant and mechanistically central annotations. These nodes can denote either mutation/genetic evidence, specific functional roles in the pathway, central mechanistic hubs, or heavy literature support.

These pathways were mapped in Qiagen IPA (v.01-22-01; QIAGEN Inc., Germantown, MD, USA, https://digitalinsights.qiagen.com/IPA, accessed 16 December 2025) software to identify the differentially expressed genes (DEGs) and predicted activation/suppression involved in each pathway. The results show NPC86-mediated stabilization of Gas5 affecting a cascade of genes in these pathways. Supplemental Table S5 lists these top pathways with the involved DEGs. In the Parkinson’s disease (PD) pathway (Figure 5), the NMDA receptor was predicted to be upregulated, while the AMPA receptor was predicted to be downregulated. The NMDA receptor, a glutamate receptor pathway, is shown to be dysfunctional in the cortex of PS19 mice along with de-coupling and decreased nitric oxide (NO) production [19]. Our results show predicted activation of the NMDAR pathway and an increase in nNOS signaling with NPC86 treatment. Interestingly, the voltage-gated calcium channel was predicted to be downregulated.

**Figure 6:**
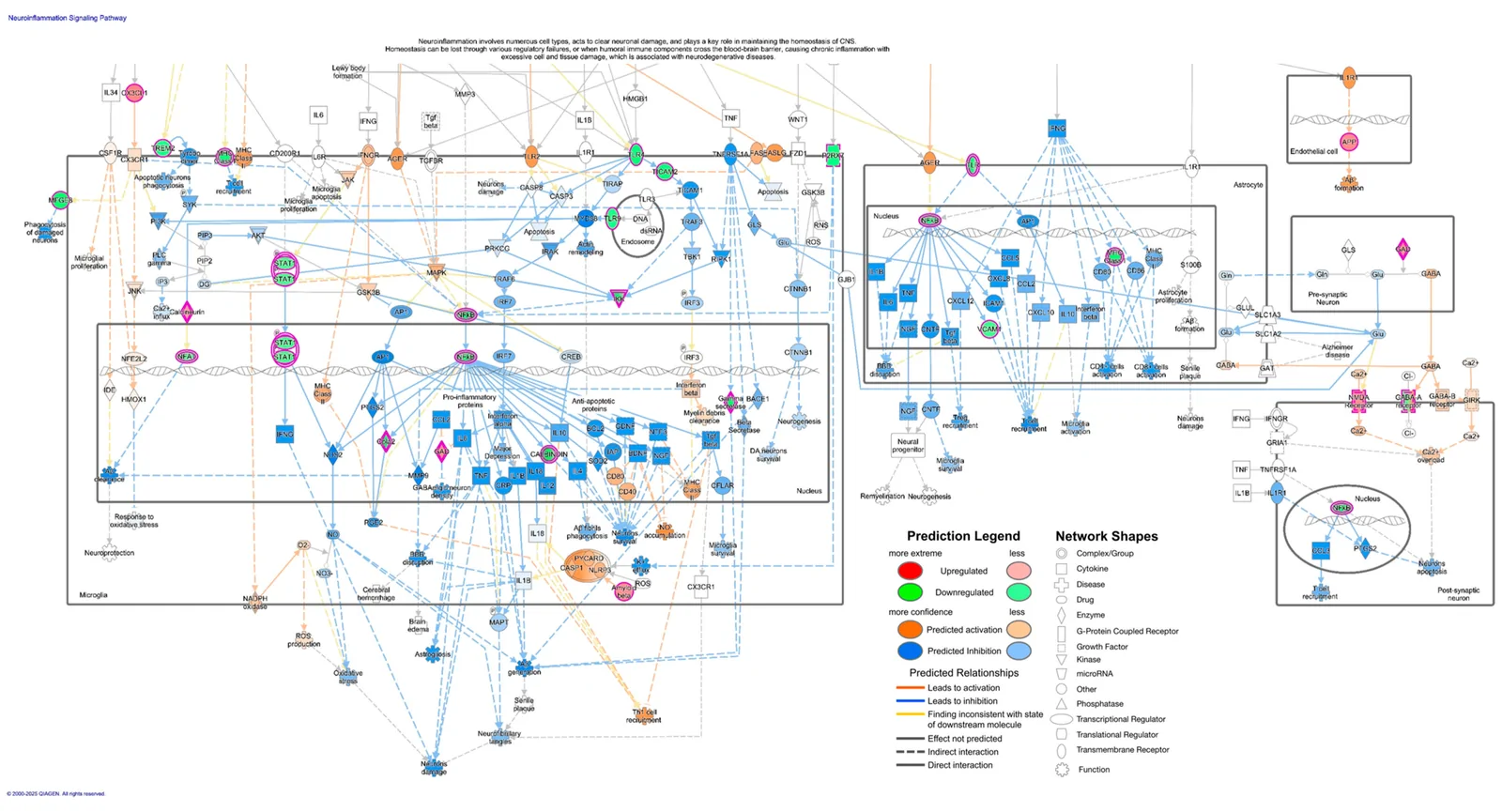
Neuroinflammation pathway generated using Qiagen Ingenuity Pathway Analysis software v.01-22-01 (QIAGEN Inc., Germantown, MD, USA, https://digitalinsights.qiagen.com/IPA, accessed 16 December 2025).

The neuroinflammation pathway (Figure 6) was significantly downregulated in the PS19 mice treated with NPC86. Here, Trem2 is predicted to be downregulated in the microglia, which inhibits apoptotic neuron phagocytosis. Furthermore, in the microglia, it is predicted that TLR4 is downregulated, which causes downregulation of IKK and NFkB. Within the microglial nucleus, the downregulation of NFkB is predicted to inhibit proinflammatory and anti-apoptotic proteins such as IL6, TNF, IL1β, IL10, BCL2, NTF3, and TGFβ, resulting in reduced blood–brain barrier (BBB) disruption, astrogliosis, and neuron damage. A similar pattern can be observed in the astrocytes, with the predicted downregulation of TLRs subsequently inhibiting BBB disruption, T reg recruitment, microglia activation, and CD4+ cell activation. Within endothelial cells, the amyloid precursor protein (APP), which plays a role in normal vasculature, endothelial cell proliferation, adhesion and migration, was predicted to be slightly upregulated with NPC86 treatment. Meanwhile, inside the pre-synaptic neuron, it is predicted that a combined upregulation of GAD and inhibition of glucose metabolism will activate GABA, which downregulates the GABA-A receptor while increasing the influx of calcium (Ca^2+^) through NMDA-R in the post-synaptic neuron.

The multiple sclerosis (MS) pathway (Supplemental Figure S5) overlaps with the PD pathway in presenting with chronic neuroinflammation, mitochondrial dysfunction and oxidative stress [20]. ORA analysis showed a predicted upregulation of NMDAR and predicted downregulation of the sodium–calcium exchange (NCX) receptor in response to NPC86 treatment. NCX normally removes Ca^2+^ along with an influx of Na^+^; however, it is shown that in an Alzheimer’s disease model with dysregulated Ca^2+^ homeostasis, there is an increase in NCX1, NCX2 and NCX3 induced by tau pathology and Aβ [21]. Thus, inhibition of the NCX receptor by NPC86 may serve as a neuroprotective pathway in tauopathy. Gα(s) signaling (Supplemental Figure S6) is predicted to activate protein kinase A (PKA), which is implicated in tau phosphorylation at S214. This is in concurrence with our Western blot data showing a decrease in pTauS214 upon NPC86 treatment. Furthermore, within microglia, it is predicted that proinflammatory cytokines and TLRs are depleted, resulting in inhibition of the neuroinflammation of the microglia. These changes contribute to ultimately inhibiting oxidative stress and Parkinson’s disease pathways in response to NPC86 treatment in PS19 mice. ORA analysis also revealed that the interferon gamma signaling pathway (Supplemental Figure S7) was significantly downregulated in the PS19 mice treated with NPC86.

**Figure 7:**
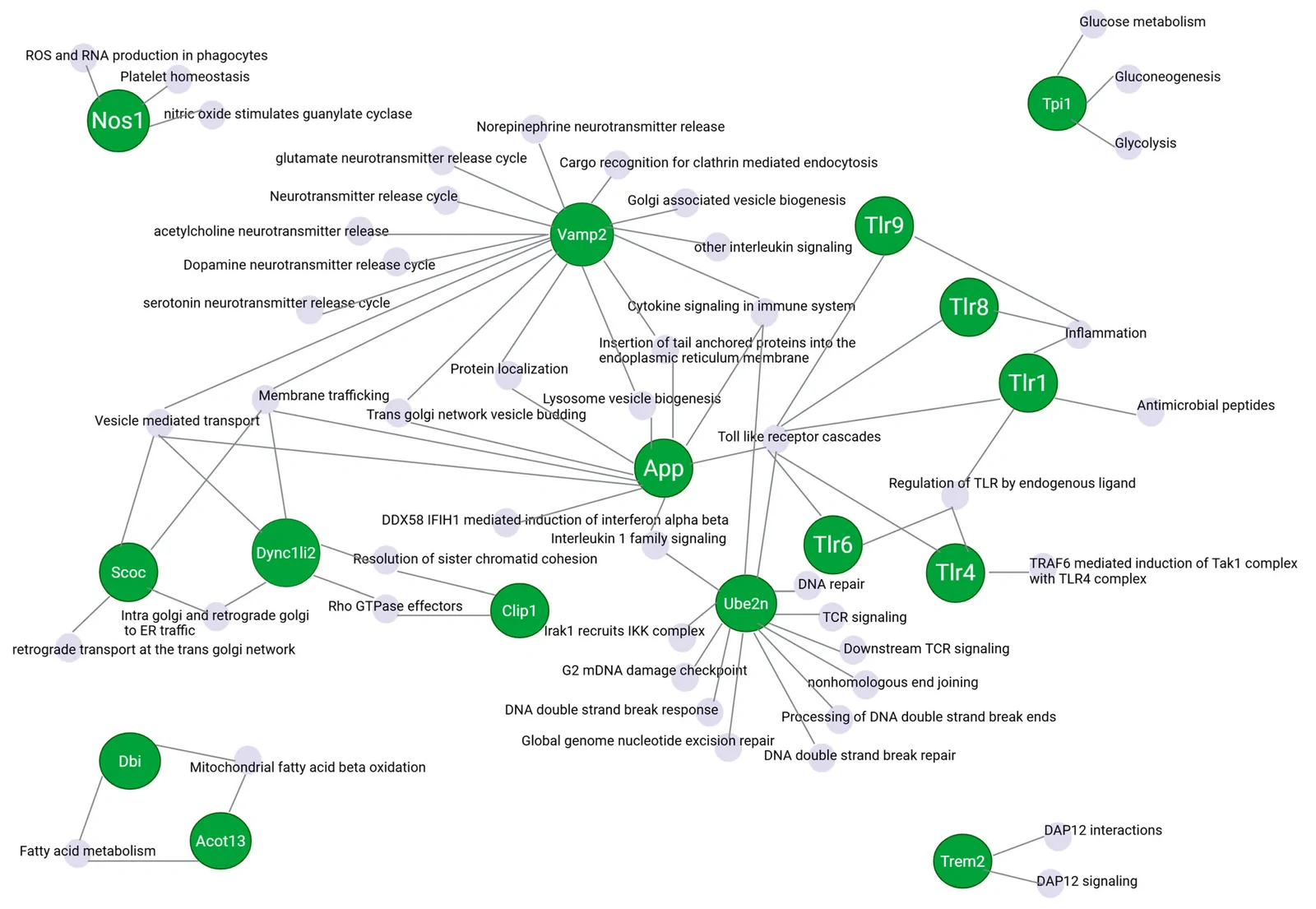
Network analysis of combined top DEGs and DEGs of interest (tauopathy model genes). Created in BioRender. Krause-Hauch, M. (2026) https://BioRender.com/lmyfs0h. Copyright 2026, Krause-Hauch, M. Publication license can be found in the Supplemental Materials. TRAF6 mediated induction of TAK1 complex within TLR4 complex.

A network analysis of the top genes and selected genes of interest was performed (Figure 7) to determine the connection between DEGs and highly significant pathways (FDR < 0.05). Vamp2 was associated with 16 pathways involved in (1) neurotransmitter release; (2) cytokine and interleukin signaling; (3) vesicle transport, biogenesis, and budding; (4) membrane trafficking; and (5) protein localization. App was associated with 10 pathways, 7 of which were common with Vamp2 (vesicle transport, budding, and biogenesis; cytokine signaling; protein localization; insertion of tail-anchored proteins). Other pathways App is associated with include toll-like receptor cascades, interleukin 1 family signaling, and DDX58 IFIH1-mediated induction. Ube2n is associated with toll-like receptor cascades and interleukin 1 family signaling, along with App and cytokine signaling in the immune system with both App and Vamp2. Ube2n is also associated with multiple pathways involved with TCR signaling and DNA damage and repair. Scoc is shown to play a role in vesicle-mediated transport, membrane trafficking, and trans-Golgi transport. Dync1li2 is also shown to function in vesicle-mediated transport, membrane trafficking, and trans-Golgi transport, in addition to the resolution of sister chromatid cohesion and rho GTPase effector pathways along with Clip1. The TLRs (1, 4, 6, 8, and 9) play a role in the toll-like receptor cascade pathway. TLR1, 8, and 9 are also involved in the inflammation pathway. TLR1, 4, and 6 are associated with the regulation of TLR by the endogenous ligand. TLR1 also plays a role in antimicrobial peptides, while TLR4 in involved with the TRAF6-mediated induction of Tak1. Dbi and Acot13 are involved in fatty acid metabolism and mitochondrial fatty acid beta oxidation. Nos1 is associated with ROS and RNA production in phagocytes and platelet homeostasis, and nitric oxide stimulates guanylate. Tpi is found in glucose metabolism, gluconeogenesis, and glycolysis pathways. Finally, Trem2 was found in DAP12 interactions and signaling pathways.

### 2.6. Disruption of Dysregulated NMD-Mediated Gas5 Turnover Decreases Neuroinflammation

**Figure 8:**
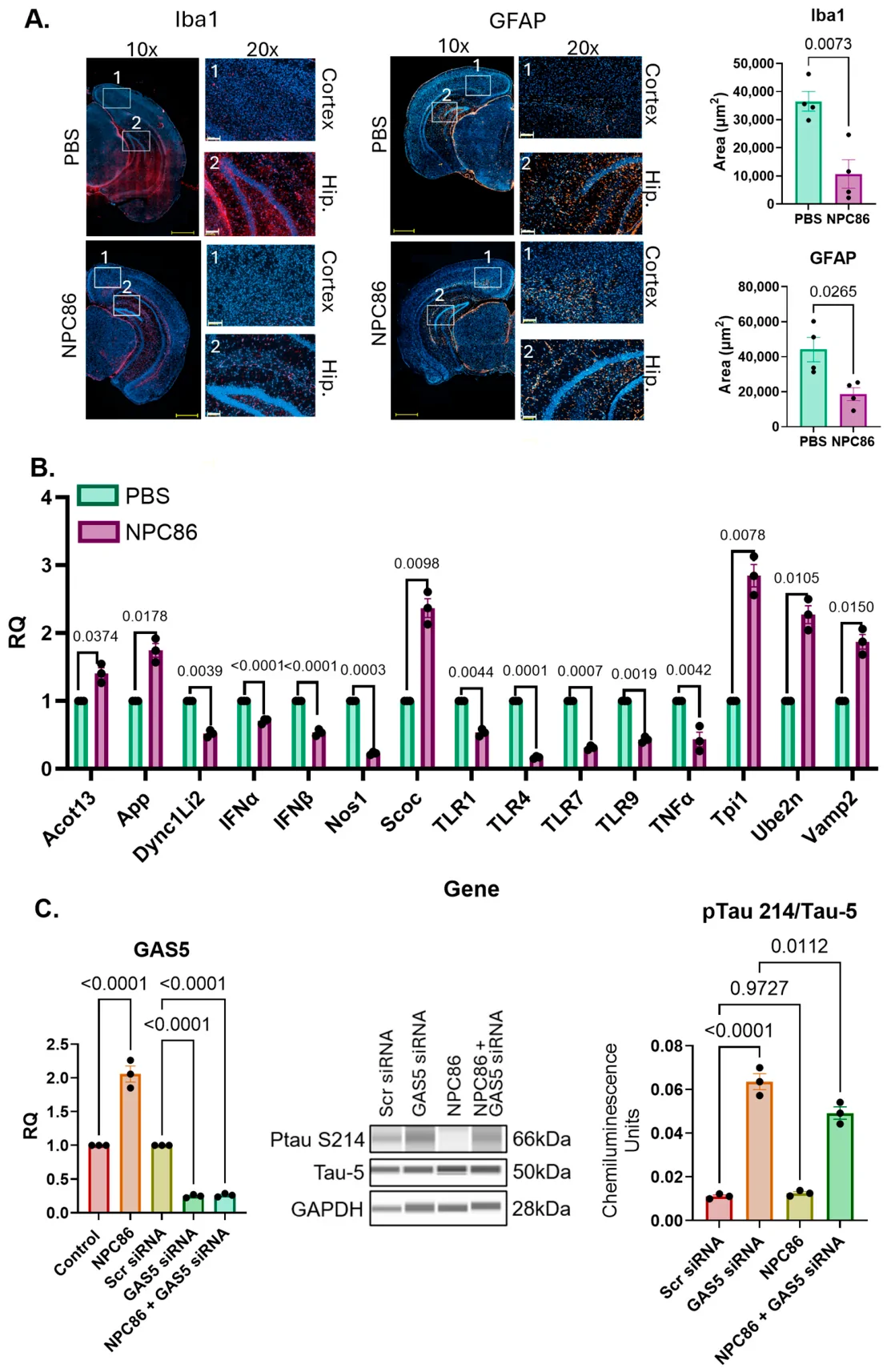
(**A**) Immunofluorescence staining was performed with DAPI counterstain (blue) to visualize Iba1 (red) and GFAP (orange) abundance in the brain at 10× (scale bar = 1 mm) and 20× (scale bar = 100 µm). The graph shows quantification of Iba1 or GFAP abundance. Four tissue samples per group were analyzed. The average area of Gas5 abundance was quantified from 12 equally sized fields, which were equally distributed across each tissue section. *n* = 4 mice. Statistical analysis performed using unpaired Student’s t-test. Green bars represent PBS, and purple bars represent NPC86. In the 10× images, the numbered boxes correspond to the brain region in the 20× images: 1 corresponds to the cortex, and 2 corresponds to the hippocampus. (**B**) Cortex samples from PS19 mice treated with NPC86 or PBS were used in SYBR Green qPCR to validate the expression of genes identified in the DSP analysis and additional inflammatory markers. Statistical analysis using unpaired Student’s *t*-test. Green bars represent PBS, and purple bars represent NPC86 (**C**) HEK293 cells over-expressing mutant tauP301L were treated with NPC86 for 24 h, followed by transfection with either scrambled siRNA or GAS5 siRNA for 24 h. Whole-cell lysates were analyzed with qPCR for Gas5 expression (left panel) and automated Western blotting (Jess) for pTau S214 and Tau-5 normalized to GAPDH (middle and right panels). Immunoblotting was analyzed using the ProteinSimple Jess built-in software, Compass for Simple Western v.7.0.0. The image shows the pseudo-blot (lane view) image generated for each antibody (as indicated in the figure) by Compass software from the capillary signals. Jess measures total protein in every capillary and normalizes the signal against actual protein load, reported as chemiluminescent units. Next, each antibody’s chemiluminescent units were normalized to the Gapdh chemiluminescent units. pTauS215/Tau-5 is plotted on the graph. *n* = 3. Statistical analysis was performed using one-way ANOVA. In the left panel, the red bar represents the control, the orange bar represents NPC86 treatment, the yellow bar represents treatment with scrambled siRNA, the green bar represents treatment with GAS5 siRNA, and the blue bar represents treatment with NPC86 followed by GAS5 siRNA. In the right panel, the red bar represents treatment with scrambled siRNA (control), the orange bar represents treatment with GAS5 siRNA, the yellow bar represents treatment with NPC86, and the green bar represents treatment with NPC86 followed by GAS5 siRNA.

Activated microglia and astrocytes are significant contributors to neuroinflammation. Immunohistochemistry results (Figure 8A) showed a decrease in Iba1 staining, indicating a decrease in the microglial burden in the brain after NPC86 treatment. GFAP staining showed decreased activated astrocytes with NPC86 treatment. These results demonstrate NPC86 treatment decreases proinflammatory microglia and activated astrocytes.

### 2.7. Validation of DSP Results

To confirm the DSP results, RT-qPCR analysis was performed on genes that were significantly differentially expressed in NPC86 compared to PBS-treated PS19 mice. The results (Figure 8B) confirmed the expression patterns observed in the DSP analysis. Acot13, App, Scoc, Tpi1, Ube2n, and Vamp2 mRNA levels were significantly increased in mice treated with NPC86. Dync1Li2 and Nos1 mRNA levels were decreased in NPC86-treated mice. Since the DSP results had shown a decrease in neuroinflammation, we evaluated the expression of IFNα, IFNβ, TLRs and TNFα using RT-qPCR. The results demonstrate a decrease in these neuroinflammatory genes in response to NPC86 treatment.

### 2.8. Validation of Gas5-Dependent Outcome of NPC86 Treatment

We confirmed the specificity of NPC86 using HEK293 cells over-expressing mutant tauP301L (gift from Dr. Laura Blair, USF). Cells were treated with NPC86 for 24 h, followed by transfection with either scrambled siRNA or GAS5 siRNA for 24 h (previously validated from three siRNAs for the absence of non-target effects). Whole-cell lysates were analyzed with automated Western blotting (Jess; BioTechne, Minneapolis, MN, USA) for pTau S214, which was identified as significantly reduced by NPC86 treatment in the cortex of PS19 mice (Figure 3A above). The results (Figure 8C) demonstrate that NPC86 decreases the phosphorylation of TauS214, which is abolished by the depletion of GAS5. Full blots can be found in Supplemental Figure S4. These results validate that NPC86 significantly decreased tau S214 phosphorylation in a GAS5-dependent manner.

## 3. Discussion

While the NMD pathway is well studied in other organs, less is known about the role of NMD pathway’s target genes in neurodegenerative diseases and specifically in tauopathies. Hence, we evaluated Gas5, an NMD-target gene, in a tauopathy mouse model and elucidated the effects of stabilizing Gas5 levels by inhibiting NMD-mediated transcript turnover. We used NPC86, an RNA-targeting small molecule, as a molecular tool to examine the mechanisms. The binding kinetics, distribution, specificity and safety of NPC86 was established in vivo previously [10]. Our results demonstrate that disrupting the NMD pathway resulted in increased levels of Gas5 and slowed the progression of age-related neurodegenerative pathways in PS19 mice. Interestingly, we observed that female PS19 mice did not respond to NPC86, while the male PS19 responded significantly. This sex-dependent response to rescue by NPC86 in PS19 mice is currently being evaluated. The results in this study describe the response of male PS19 mice to the NPC86-mediated increase in Gas5 levels in the brain.

Noncoding transcripts such as lncRNAs, miRNA and snoRNA are regulated predominantly by NMD to maintain their physiological levels, dependent on their role in the organ [2]. In disease states, NMD may be protective by degrading transcripts that produce toxic products or may be detrimental by increasing the turnover of transcripts of pivotal ncRNAs. NMD regulates neuronal development [4] as well as axonal migration [5] and synaptic protein levels [6,7]. Upf1 binds to the transcript and is a central initiator protein in the NMD pathway [3]. It is established that Gas5 levels are regulated by NMD [13,14]. Our results show that endogenous Upf1 levels were not significantly different between PS19 and Ntg mice. However, PS19 mice had a significantly higher binding of Upf1 with Gas5, which was disrupted with NPC86 treatment, concurrent with increased Gas5 levels. We evaluated the levels of Gas5 in the PS19 mice as they aged, and the results show low Gas5 levels are an early event in the pathology. Thus, inhibiting NMD-mediated Gas5 turnover provides the advantage to allow titration of Gas5 to its physiological levels to re-instate its multiple functions in the brain.

Gas5 is a multi-faceted lncRNA. Its stem-loop structures allow binding to miRNAs, proteins, DNA or RNA at distinct cis-elements. It functions as a riborepressor to sequester the glucocorticoid receptor (GR) and regulate inflammatory pathways [22], a sponge for miRNAs in cancers [23] and a regulator of insulin signaling pathways [10]. This study evaluates the PS19 mouse model of tauopathy and the interconnection between brain Gas5 and neurodegeneration associated with this tauopathy. Other studies have evaluated the role of Gas5 using mouse models of genetic mutations of Alzheimer’s disease, Parkinson’s disease and other neuronal models. Chanda et al. [24] reported that Gas5 levels were significantly decreased in 5XFAD, an Alzheimer’s disease mouse model of human APP and PSEN1 transgenes with five associated mutations. They further demonstrated that Gas5, as a ceRNA, functions as an activity-responsive scaffold for mediating cAMP-dependent synaptic plasticity and transmission in dendrites. Our work aligns with that of Chanda et al., as we show that Gas5 levels were decreased in the PS19 tauopathy model. However, Zeng et al. [25] report upregulation of Gas5 levels in the 5XFAD mouse model and binding to microRNA-23b-3p to regulate the GSK3β pathway. Separately, Zhao et al. [26] showed that Gas5 increases the expression of choline acetyltransferase in PC12 neuronal cells, while Wu et al. [27] showed that Gas5 levels are associated with neuronal damage in the hippocampus of mice with depressive-like behaviors by targeting early growth response gene 1 (EGR1). In Parkinson’s disease models, Gas5 is shown to promote microglial inflammatory response [28] or promote neuronal injury by binding to miR-150 [29]. Interestingly, low serum Gas5 levels are proposed as a biomarker for PD [30]. Some of these conflicting results may be explained by evaluation of different brain regions, measuring Gas5 levels in patient blood samples, or using different mouse models of these diseases. Additionally, there are more than 27 alternatively spliced transcripts of Gas5, and different groups use primers amplifying different splice variants in PCR. Over the years, to maintain reproducibility of data, we have consistently used primers to exon 12, which is highly conserved between mouse and human and is present in all splice variants. Separately, we have shown the ratio of Gas5 within cells and its secretome varies with the disease state [10]. Results from our RNA-FISH/IF analysis showed that Gas5 colocalized with tau in PS19 mice was disrupted with NPC86 treatment. Since this was concomitant with a decrease in pTauS214 and pTau202/205, it suggests that Gas5 may function as a scaffold to aid the association of tau and kinases such as PKA and Fyn, which phosphorylate tau at these residues. NPC86 treatment increased pAKT, demonstrating that rescuing dysregulated insulin signaling alleviates early determinants of neurodegenerative diseases.

DSP analysis was used to narrow down the regions of interest to analyze the proteomic changes in response to NPC86 treatment. The top differentially expressed genes (DEGs), also verified by qPCR, increased in the cortex with the NPC86 treatment compared to PBS, including proteins involved in synaptic function and vesicle transport (Vamp2, Arl4d), protein turnover and stress response (Dbi, Ube2n), mitochondrial health and energy metabolism (Acot13, Tpi1, Timm17a), and cytoskeleton dynamics (Tmsb10) and intracellular signaling (Scoc, Tmem181a). The vesicle-associated membrane protein 2 (Vamp2) is a component of synaptic vesicles, and its levels are decreased in patients with AD, and it is correlated to decrease in cognition [31,32]. Our results show that Vamp2 is low in PS19 mice, and treatment with NPC86 increased its levels significantly. This suggests that Gas5 may facilitate the fusion of synaptic vesicles with the neuronal membrane and formation of the SNARE complex via regulation of Vamp2 levels. Interestingly, it has been previously shown that α-synuclein associates with Vamp2 at the synapses to prevent aggregation of α-synuclein [33,34]. Abnormal aggregation of α-synuclein is a hallmark of Parkinson’s disease. The DSP results from this study also show that pathways in Parkinson’s disease were downregulated with NPC86 treatment. NPC86 treatment increased levels of diazepam binding inhibitor (Dbi; also known as Acyl-CoA binding protein), which functions as an endogenous modulator of the GABA-A receptor, aids the suppression of abnormal neuronal excitability such as in seizures and promotes plasticity in neurogenesis [35,36]. However, if Dbi is over-expressed it can impair hippocampus-dependent learning [37], thus pointing to its role in a careful balance of neurometabolic processes. Ubiquitin-conjugating enzyme 2 UBE2N (also known as Ubc13) acts along with E3 ligase Parkin to mediate mitophagy, resulting in the autophagic destruction of the damaged mitochondria seen in Parkinson’s disease [38]. NPC86 treatment increased Ube2n, suggesting an increase in the destruction of depolarized mitochondria. Triosephosphate isomerase (TPI1) is an essential glycolytic enzyme crucial for providing brain ATP [39]. TPI1 levels are decreased in AD and schizophrenia [40,41]. Our results show that NPC86 treatment increased TPI1 levels. Gas5 regulates PI3K/Akt signaling [10,42], and it is separately shown that TPI1 activates the Akt pathway [43]. These results suggest that stabilizing Gas5 levels with NPC86 promotes neuronal PI3K/Akt signaling pathways, providing energy (ATP) for neurotransmitter release, fuels for metabolism and ion pumps and neuromodulation. Acyl-CoA thioesterase 13 (Acot13) is present in the mitochondria of highly oxidative tissues (such as the brain), and it is involved in functions associated with lipid biosynthesis, gene transcription, and signal transduction and plays a role in energy metabolism [28]. DSP analysis and qPCR validation found that Acot13 levels increased with NPC86 treatment. A previous study reported that Acot13 was downregulated in the brains of AD mice compared to young mice [44]. Acot13 has been investigated in other disease systems such as ovarian cancer and kidney disease, but currently there are no known studies investigating the role of Acot13 in tauopathies. Trem2, a microglia-specific receptor for lipids, APOE and Aβ, regulates the innate immune function, and recent studies have shown its role in the activation of microglia in promoting Alzheimer’s disease [45]. However, other groups [46] using Trem2-/- mice showed attenuated tau pathology, suggesting that reduced Trem2 impairs the capacity of microglia to contribute to tau spreading. Our results show that NPC86 treatment decreased Trem2 levels along with a decrease in the pro-neuroinflammatory toll-like receptor family and Caspase-9, an initiator protease in tauopathy and Alzheimer’s disease (secondary tauopathy) brains. Results demonstrate that NPC86 treatment in PS19 mice increased NMDAR. Previously, Jos et al. [47] showed that in normal conditions, tau binds to Fyn to target it to the post-synaptic compartment and promote the influx of Ca^2+^ through NMDAR. In tauopathies, phosphorylation of tau at S214 inhibits its binding to Fyn, promotes tau aggregation and impairs the glutamatergic synaptic transmission of Ca^2+^ through NMDAR. Our results shown here demonstrate that NPC86 treatment decreased pTauS214, suggesting alleviation of the detrimental effects of pathological tau.

### Limitations

The PS19 mouse model has high variability across individual mice, and thus analysis of some DEGs and pathways did not show high significance. In this study, we did not evaluate tau seeding or perform an in-depth evaluation of the role of NMD and Gas5 in oligodendrocytes, astrocytes and microglia. We did not see a significant response to NPC86 in female PS19 at 9 months, and this sex difference outcome will be pursued in future studies. Future studies will include behavior and DSP in mice treated with NPC86 starting at 5 months of age up to 10 months, when the tauopathy is prominent.

## 4. Materials and Methods

### 4.1. Animals

Equal male and female PS19 transgenic mice (hemizygous for Tg(Prnp- MAPT*P301S)PS19Vle) and non-transgenic (Ntg; non-carrier for Tg(Prnp-MAPT*P301S)PS19Vle) littermates were purchased from Jackson laboratory. PS19 mice express the human microtubule-associated protein tau (MAPT) mutant, P301S, at five-fold higher than the endogenous mouse MAPT protein. PS19 mice begin to exhibit physiological signs of tauopathy at three months of age, which will significantly progress by 10 months of age [14,15,16,17]. These signs include neuroinflammation, motor deficits, and cognitive impairment. The James A. Haley Veteran’s Hospital and University of South Florida Institutional Animal Care and Use Committee (IACUC) approved all experimental procedures with animals consistent with the governing guidelines and recommendations of AWA and HREA. All experiments complied with the ARRIVE guidelines. All animals were raised and studied in pathogen-free environments and housed in plastic, sawdust-covered cages, with a normal light–dark cycle and free access to chow and water.

### 4.2. NPC86 Treatment

**Figure 9:**
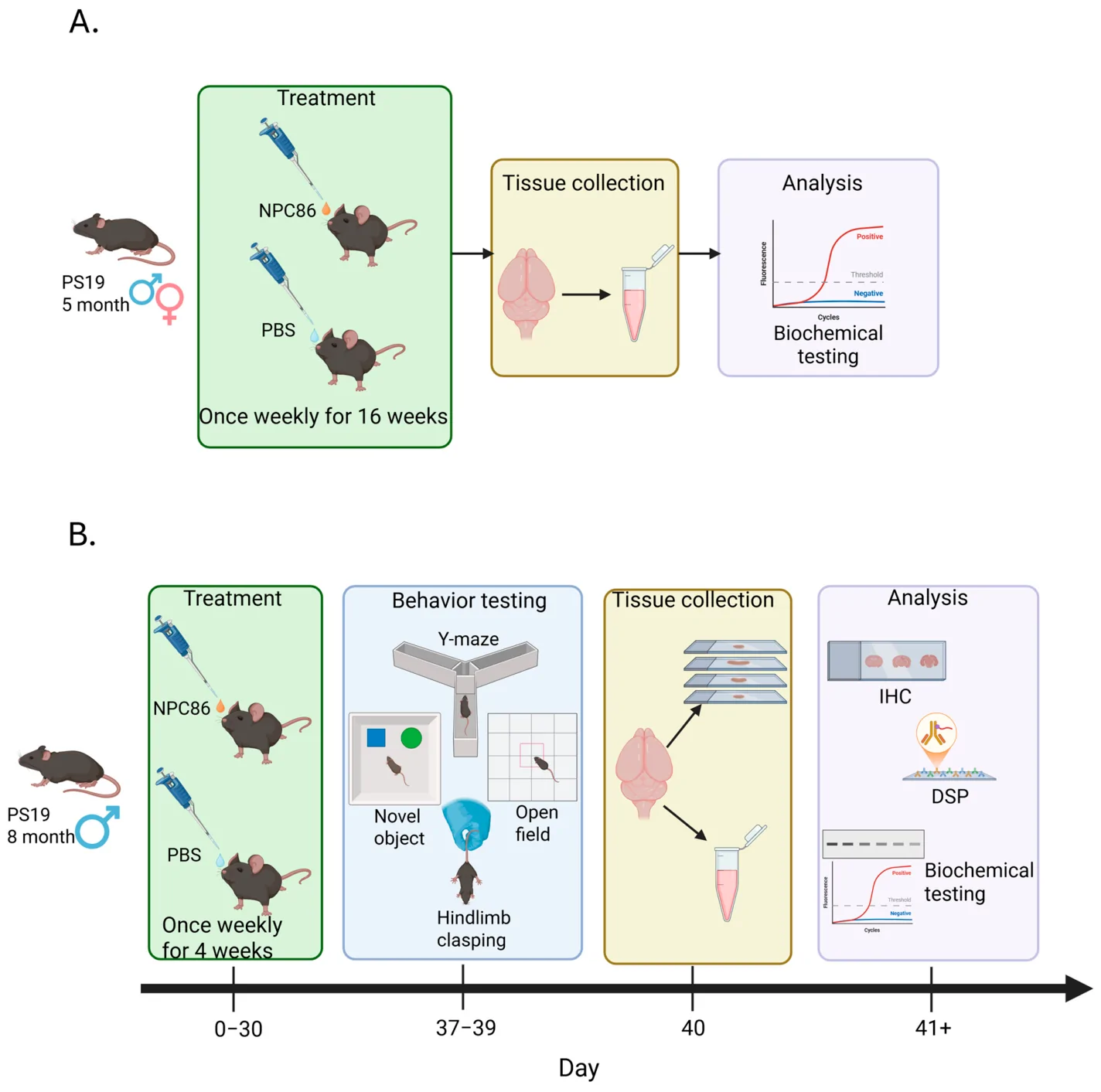
(**A**) Schematic of group 1 mouse experiments. Created in BioRender. Krause-Hauch, M. (2026) https://BioRender.com. Copyright 2026, Krause-Hauch, M. Publication license can be found in the Supplemental Materials. (**B**) Schematic of group 2 mouse experiments. Created in BioRender. Krause-Hauch, M. (2026) https://BioRender.com/k64ep35. Copyright 2026, Krause-Hauch, M. In both panels, green boxes indicate the treatment phase, blue boxes represent behavior testing, yellow boxes represent tissue collection, and purple boxes represent analysis.

For the group 1 study, five-month-old PS19 were administered NPC86 for 4 months, and for the group 2 study, eight-month-old PS19 were administered NPC86 for 1 month. Power analysis for the number of mice per group required to detect a difference of 20% was determined to be 12 at a power of 0.9 based on the known variances and the anticipated group mean differences (α = 0.05, β = 0.9, and SD = 20%). To account for immunohistological and biochemical measures, which require extra samples, 18 mice were included in each group. Mice were randomly assigned to biochemical analysis (*n* = 12/group) or immunohistochemistry (IHC) (*n* = 6/group), and blinded IDs were generated. NPC86 was administered intranasally, as described previously by our lab [10]. Briefly, awake mice were held in a supine position with the neck in extension, and 5 μL of NPC86 was administered using a pipette tip in alternating nostrils, with a 10–15 s pause between nostrils (10 μL total volume). At 5 months of age, the group 1 mice were given 100 ng of NPC86 every 7 days for 4 months (Figure 9A). The group 2 mice started treatment at 8 months and were given 200 ng of NPC86 every 7 days for 4 weeks (Figure 9B). Euthanasia was at 9 months for both groups, at which point, the brains were collected for analysis.

### 4.3. Behavior Testing

One week after the last NPC86 dose was administered, the mice underwent behavioral testing to establish cognitive function and the memory effects of receiving NPC86 treatment. Y-maze, novel object recognition, open field testing, and hindlimb clasping were performed as described below. For each test, the mice were acclimated to the testing room for 30 min prior to testing. All equipment was cleaned with 70% ethanol prior to the start of testing and after each trial. *n* = 12 animals per group underwent behavioral testing.

Hindlimb clasping: Mice were removed one at a time from their home cage, gently grasped approximately 1.5″ from the base of the tail, and suspended in the air for about 5 s with the abdomen facing the tester. The positioning of the hindlimbs was observed, and then the mouse was returned to the home cage. The mice were scored on a scale of 0–3 based on the following parameters: (0) both hindlimbs extend fully outwards with free movement; (1) one or both hindlimbs are partially or fully retracted towards the abdomen for more than 2 s; (2) one or both hindlimbs are partially or fully retracted towards the abdomen for more than 4 s; and (3) both hindlimbs are fully clasped against the abdomen for the duration of the observation period without moving.

Y-maze: A Y-maze from Stoelting Co. (Stoelting Co., Wood Dale, IL, USA; catalog #60180) was used for testing. After habituation, each mouse was placed at the same end of one arm (designated arm “B”) and allowed to move freely through the maze for 8 min. The full duration of each trial was recorded. The footage was later analyzed with ANY-maze software (v7.33; Stoelting Co., Wood Dale, IL, USA) to determine the total number of alterations (times a mouse entered the three arms of the Y-maze without returning to a previously entered arm) and the total number of entries into each arm.

Novel Object Recognition Test: Testing took place over 3 days. On the first day, the mice were accustomed to the testing apparatus, and the open field test was conducted. Each mouse was placed in the center of the arena, allowed to freely explore for 15 min, and then returned to its home cage. On the second day (24 h after the habituation period), the mice were trained by placing two identical objects in opposite quadrants of the area. Each mouse was placed in the center of the arena, equidistant from the two objects, and allowed to freely explore for 10 min. On the third day (24 h after the training period), one object used during training (familiar object) and one novel object were placed in opposite quadrants of the arena in the same location used during training. Each mouse was again placed in the center of the arena, equidistant from the two objects, and allowed to freely explore for 10 min. For all 3 days, the full duration of each trial was recorded. The footage was analyzed with ANY-maze software (v7.33; Stoelting Co.) to measure exploration for the familiar and novel objects. The NOR discrimination index was calculated as [(time spent exploring novel object) − (time spent exploring familiar object)]/(total exploration time).

Open Field: Each mouse was placed in the center of the arena, allowed to freely explore for 15 min, and then returned to its home cage. The full duration of each trial was recorded. The footage was analyzed with ANY-maze software (v7.33; Stoelting Co.).

### 4.4. Droplet Digital PCR (ddPCR)

To evaluate the number of GAS5 copies present per ng RNA, droplet digital PCR (ddPCR; Bio-Rad Laboratories, Hercules, CA, USA) was performed. RNA was isolated from frozen cryogenic ground tissue using RNAzol^®^ RT (Molecular Research Center, Inc., Cincinnati, OH, USA). The RNA concentration was quantified using Nanodrop and stored at −20 °C in DPEC water. Then, 500 ng of RNA (260/230 > 1.8 and 260/290 > 1.8) was used to synthesize cDNA using an iScript™ cDNA Synthesis Kit (Bio-Rad, #1708891). A 1:100 cDNA solution was synthesized prior to droplet generation via the QX200 Droplet Generator (Bio-Rad Laboratories, Hercules, CA, USA). DNA was then partitioned into 20,000 droplets for amplification using QX200 ddPCR EvaGreen Supermix. Primer information for Gas5 is included in Table S1. Each droplet was analyzed for Gas5 gene copies using the QX200 droplet reader, and results were analyzed with QuantaSoft™ Analysis Pro version 1.7.4.0917 (Bio-Rad). The average minimum and maximum Poisson Confidence was 64.5 and 73.0, respectively. The average number of accepted droplets was 17,040.86, with an average of 961.6 positives and 16,079.2 negatives. The average sample amplitude was 2121.5. The copies per well were divided by the amount of RNA used, and copies per ng RNA was calculated. Our final reported concentration is in Gas5 copies/ng RNA.

### 4.5. qPCR

RNA was isolated using RNAzol^®^ RT (Molecular Research Center, Inc.), and 1 µg of RNA (260/230 > 1.8 and 260/290 > 1.8) was used to synthesize cDNA with the iScript™ cDNA Synthesis Kit (Bio-Rad, #1708891). The target was amplified with SYBR™ Green Universal Master Mix (Applied Biosystems, Waltham, MA, USA, #4309155) and respective primers, including specifically designed GAS5 primers to amplify exon to measure total GAS5 levels. Primer concentrations were optimized for a single melt curve and consistent amplification. qPCR was performed on the ViiA 7 Real-Time PCR System (Applied Biosystems). Samples were run in triplicate with a standard series, no template control, and no reverse transcriptase control. A standard curve was generated for each target and used to calculate the absolute quantities (AQ) of target expression, which was then normalized to U6 (Gas5) or β-actin (all other genes). The relative quotient (RQ) was determined using the comparative method (ΔΔCT). Primer sequences are shown in Table S1. *n* = 10 samples were used in qPCR analysis.

### 4.6. Immunohistochemistry (IHC)

Histology: *n* = 6 brains per group were sectioned, stained, and analyzed for RNAscope, RNA-FISH/IF and H&E, as described below. Mouse brains were perfused with 4% PBS/paraformaldehyde solution, fixed in formalin, and subsequently processed and embedded in paraffin wax. Then, 5 µm sections were cut using a microtome, mounted on SuperFrost Plus microscope slides and baked in a dry oven for 1 h at 60 °C to improve sample adhesion to the slide. A commercial Hematoxylin and Eosin (H&E) staining kit was purchased from Abcam (Cambridge, UK; Cat #ab245880). Staining was completed according to manufacturer instructions on de-paraffinized sections. Slides were imaged using a Keyence BZ-X800 microscope (Keyence, Itasca, IL, USA).

RNAScope: Coronal brain slices were permeabilized through an ethanol series. The sections were stained using an RNAscope GAS5 probe (ACD kit #561630, Newark, CA, USA) using Akoya Opal secondary fluorophores as per the manufacturer’s instructions. The GAS5 probe was hybridized, and the signal was amplified using the RNAscope AMP reagent, followed by labeling with Opal fluorophores. Slides were imaged using the Keyence BZ-X800 (Keyence, Itasca, IL, USA) microscope and quantified with the BZ-X800 Analyzer software (v1.1.1.8; Keyence, Itasca, IL, USA). Statistical analysis was performed using Student’s t-test of the average area (µm^2^) of Gas5 fluorescence of 12 equally sized fields uniformly distributed across each tissue section.

RNA-Fluorescent In Situ Hybridization with Immunofluorescence (RNA-FISH/IF): Coronal brain slices were deparaffinized and rehydrated with a series of washes at room temperature: xylene for 3 min (2×), 1:1 xylene/100% EtOH for 3 min, 100% EtOH for 3 min (2×), 95% EtOH for 3 min, 70% EtOH for 3 min, 50% EtOH for 3 min. Slides were then rinsed in running tap water. Antigen retrieval was performed by immersing slides for 15 min in 1× sodium citrate buffer (pH 6.0) at 95 °C. Probe hybridization with Gas5 ex12 was performed with the HCRTM Gold RNA-FISH kit (Molecular Instruments, Los Angeles, CA, USA) according to manufacturer’s instructions. After probe hybridization, IHC was performed with a primary antibody cocktail prepared with total Tau-5 mouse mAb, with Alexa Fluor 546 anti-mouse secondary antibody. *n* = 4 brains per group were sectioned and stained. Twelve ROIs per mouse were analyzed, and the average was calculated for each mouse. To evaluate colocalization of Gas5 and Tau-5, Mander’s overlap coefficient was calculated using R package colocr (v.0.1.1) [48].

A separate IHC was performed with a primary antibody cocktail of Iba1 goat mAb and GFAP rabbit mAb, with Alexa Fluor 647 donkey anti-goat and Alexa Fluor 594 goat anti-rabbit secondary antibodies. Slides were imaged with a Keyence BZ-X800 fluorescence microscope (Keyence, Itasca, IL, USA) and quantified with the BZ-X800 Analyzer software (v1.1.1.8; Keyence, Itasca, IL, USA). Statistical analysis was performed using Student’s t-test of the average area (µm^2^) of fluorescence of 12 equally sized fields uniformly distributed across each tissue section. Detailed information regarding the antibodies can be found in Table S2.

### 4.7. DSP ROI Collection and DSP Analysis

Coronal brain slices were deparaffinized and rehydrated through a series of washes at room temperature: xylene for 5 min (3×), 100% EtOH for 5 min (2×), 95% EtOH for 5 min (2×), and ddH_2_O for 5 min (2×). Antigen retrieval was performed by immersing slides for 15 min in 1× sodium citrate buffer (pH 6.0) at 95 °C in a steamer. After removal from the steamer, the slides in the buffer were allowed to stand for 25 min on a lab bench, and then they were washed in 1× TBS-T for 5 min. The slides were blocked with GeoMx DSP Buffer W according to the manufacturer’s instructions, followed by overnight incubation with morphology markers in a humidity chamber. After washing 3× with 1× TBS-T for 10 min each, the slides were post-fixed with 200 µL of 4% PFA for 30 min in the humidity chamber and then washed with 1× TBS-T for 5 min (2×). Nuclei staining was then performed with SYTO 13 nuclear stain according to manufacturer’s instructions. Slides were stored in 1× TBS-T until ready to load into the GeoMx DSP for scanning and the selection of ROIs.

Raw gene counts were filtered for signal intensity, retaining genes detected above the limit of quantification (LOQ) in at least 10% of ROIs, where LOQ is equal to two standard deviations above the geometric mean of the negative control probe for each ROI. Quality filtering resulted in 10,994 genes for downstream analysis, which were then Q3 normalized. A Uniform Manifold Approximation and Projection (UMAP) plot was generated with normalized expression data using the umap packages (v0.2.8.0) with default settings. Differential gene expression between groups was performed using a generalized linear model with the lm R package v.3.6.2. Genes were considered significantly differentially expressed with *p* adjusted < 0.05 and |log2 fold change| > 0.5. Differential expression results were visualized in volcano plots and violin plots using the ggplot2 package (v3.3.6) and in heatmaps with ComplexHeatmap (v2.18). Next, cell-type deconvolution was performed with the spatialdecon Bioconductor/R package (v1.22.0) [18]. The Allen Brain Atlas scRNAseq dataset [49] was used to estimate tissue-specific cell-type abundances in our GeoMx data. Pathway analysis was performed on each ROI using single-sample GSEA from the Gene Set Variation Analysis R/Bioconductor package (v.3.23) against the Reactome database. ssGSEA calculated an enrichment score for each pathway in each ROI, and these scores were used to perform differential pathway enrichment analysis using an analogous approach to the linear modeling described above. Additional overrepresentation analysis (ORA) was performed to evaluate pathways using Qiagen Ingenuity Pathway Analysis (IPA; v.01-22-01; Qiagen, Redwood City, CA, USA). *n* = 3 samples per group were analyzed for DSP.

### 4.8. ProteinSimple Jess Automated Western Blot

Automated Western Blot was performed using the ProteinSimple Jess system (ProteinSimple, Santa Clara, CA, USA), as per the manufacturer instructions. Three mice from each group were randomly selected for analysis. The ProteinSimple 12–230 kDa Separation capillary cartridges were used for sample separation, and a 1 mg/mL sample was loaded for each antibody. The antibodies listed in Table S3 were used at a 1:10 dilution with the ProteinSimple Jess Automated Western Blot System and analyzed with the system’s built-in software, Compass for Simple Western. The image shows the pseudo-blot (lane view) image generated for each antibody (as indicated in the figure) by Compass software v.7.0.0 (ProteinSimple, Santa Clara, CA, USA) from the capillary signals. Jess measures total protein in every capillary and normalizes the signal against actual protein load, which is reported as chemiluminescent units. Next, each antibody’s chemiluminescent units were normalized to the Gapdh chemiluminescent units and plotted on the graph. The images were not processed for brightness, contrast or background adjustments by any other software. Full blots can be found in Supplemental Figures S1–S4. *n* = 3 samples per group were analyzed by Jess.

### 4.9. RNA-Immunoprecipitation (RIP) Assay

RNA-immunoprecipitation (RIP) assay was completed as previously described [13] using a RIP kit purchased from Millipore Sigma (Millipore Sigma, Burlington, MA, USA; Cat#: RIP). RIP assay was performed following manufacturer instructions on five randomly selected mice in each group. For crosslinking, tissue was incubated with 1% formaldehyde in PBS for 10 min at room temperature, followed by the addition of 0.2 M glycine. Next, 10% lysate was removed as an input sample. Immunoprecipitation was performed with 2 μg UPF1 antibody (Novus), snRNP70 antibody (Novus; positive control) or IgG antibody (negative control). RNA was purified and treated with DNAse to remove genomic DNA. Real-time qPCR was performed as described above for GAS5 and U1 RNA, the binding partner for SNRNP70. The percent input (% input) was calculated using the manufacturer’s Excel template. *n* = 5 samples per group were analyzed in the RIP assay.

### 4.10. Transient Transfection of HEK293T Cells

HEK293T cells were transfected using 2.5 μL of Lipofectamine 3000 with 1 μg of human tau 4R0N P301L plasmid (pRK5 vector backbone; gift from Dr. Laura Blair, USF) per experiment. Gas5 siRNA ID n272331(selected previously for optimal Gas5 depletion and absence of off-target effects [13]) and scrambled siRNA was purchased from Thermo Fisher. Cells were transfected with 25 nM Gas5 siRNA for 24 h.

### 4.11. Statistical Analysis

Experiments were repeated three times for biological replicates. Experimental samples were run in triplicate. Normalcy was confirmed using the D’Agostino and Pearson tests and the Kolmogorov–Smirnov test. Statistical analysis was performed using the unpaired Student’s t-test, one-way ANOVA, or two-way ANOVA using GraphPad Prism, version 10.0.0 for Windows (GraphPad Software, Boston, MA, USA). * *p* < 0.05, ** *p* < 0.01, *** *p* < 0.001, and **** *p* < 0.0001 were used as significant measures.

## 5. Conclusions

The results demonstrate that PS19 mice have dysregulated neuronal networks, and inhibiting NMD-mediated Gas5 turnover enhances neuronal homeostasis by influencing brain metabolism, synaptic vesicle transport, mitochondrial function, and cytoskeletal organization within the neural network; it also decreases neuroinflammation. We and others have shown that GAS5 levels are decreased in older individuals and in patients with Alzheimer’s disease (AD) [10,26,32,50,51]. Overall, our study establishes that NMD pathways are critical in developing tauopathies, in addition to their role in other neurodegenerative diseases [45]. The results support the potential of selectively tuning transcript turnover by NMD pathways to increase levels of pivotal lncRNAs to slow the advent of neurodegenerative diseases.

## 6. Patents

NAP and JC have US Patent No. 11,278,521 for NPC86. The patent is not yet licensed, and the authors declare no competing financial interests.

## Data Availability

All data are available in the main text or the Supplementary Materials. DSP data are uploaded to Zenodo https://zenodo.org/records/22230810 (accessed on 10 June 2026).

## Supplementary Materials

The following supporting information can be downloaded at: https://www.mdpi.com/article/10.3390/ijms27177675/s1.

## Abbreviations

The following abbreviations are used in this manuscript:

GAS5	Growth-arrest specific transcript 5
lncRNA	Long noncoding RNA
FTD	Frontotemporal dementia
NMD	Nonsense-mediated decay
Ntg	Non-transgenic
DEG	Differentially expressed genes
DSP	Digital Spatial Profiling
AQ	Absolute Quantification
RQ	Relative Quantification
NOR	Novel Object Recognition
