# Supplementary material for: LncRNA Gas5, a Target of the Nonsense-Mediated Decay Pathway in the Brain, Regulates Neuroinflammation and Neurodegenerative Disease Pathways in a Tauopathy Mouse Model
Source: Int J Mol Sci. 2026 Aug 27;27(17):7675. doi: 10.3390/ijms27177675 (PMC13565889; doi:10.3390/ijms27177675)
Supplement: Supplementary file 1 [file ijms-27-07675-s001.zip › ijms-4410091-supplementary 082426.pdf]

Figure 2B

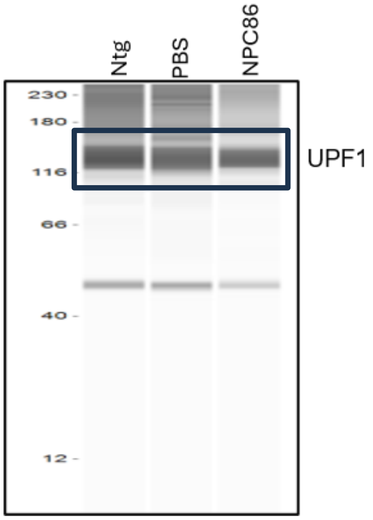


**Figure S1:** Full length ProteinSimple Jess Automated Western Blot image for UPF1 as seen in main text Figure 2B.

Figure 3A

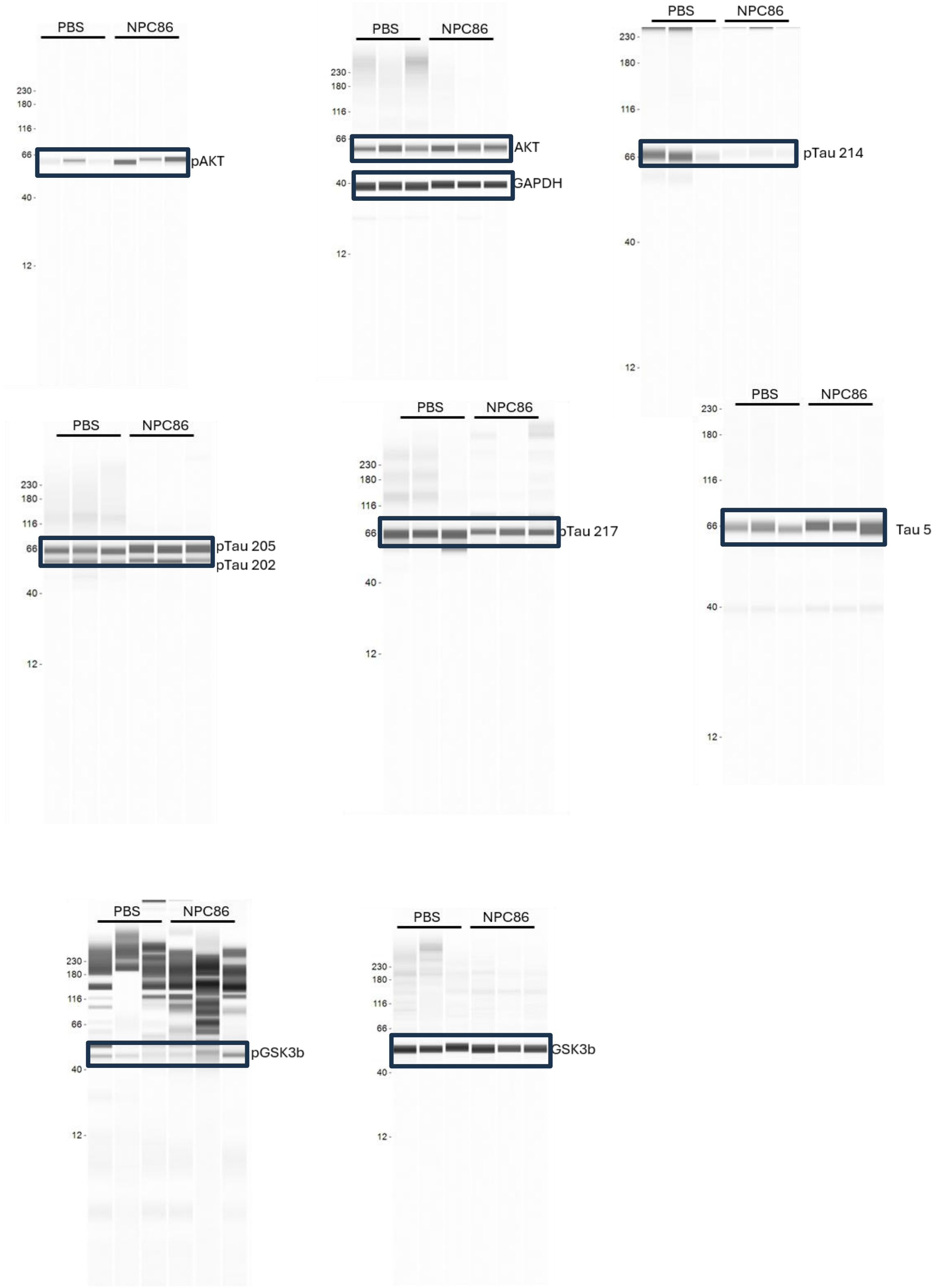


**Figure S2:** Full length ProteinSimple Jess Automated Western Blot images as seen in main text figure 3A.

Supplemental Figure S3

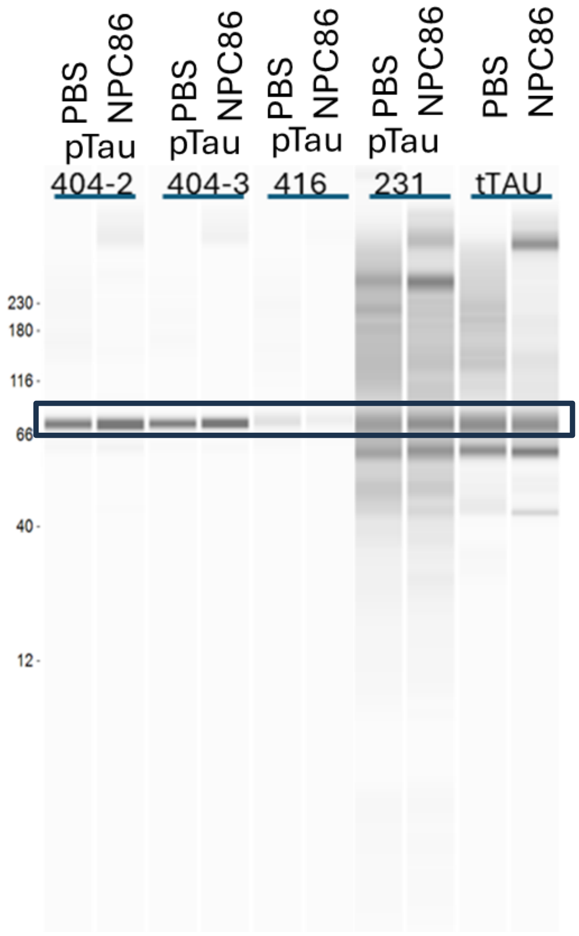


**Figure S3:** Full length ProteinSimple Jess Automated Western Blots.

Figure 8C

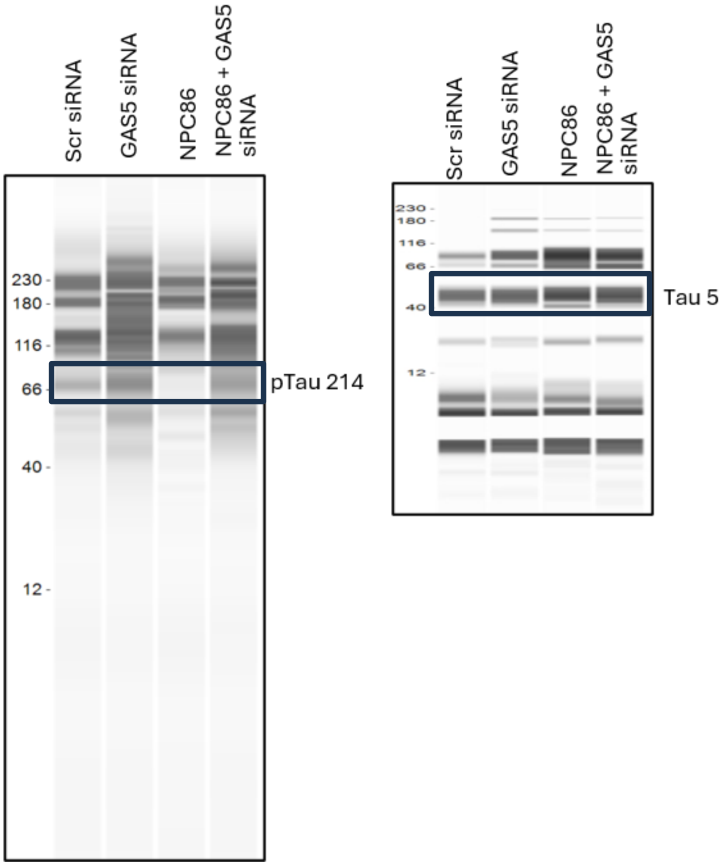


**Figure S4:** Full length ProteinSimple Jess Automated Western Blot images for pTau 214 and Tau5 as seen in main test Figure 8C.

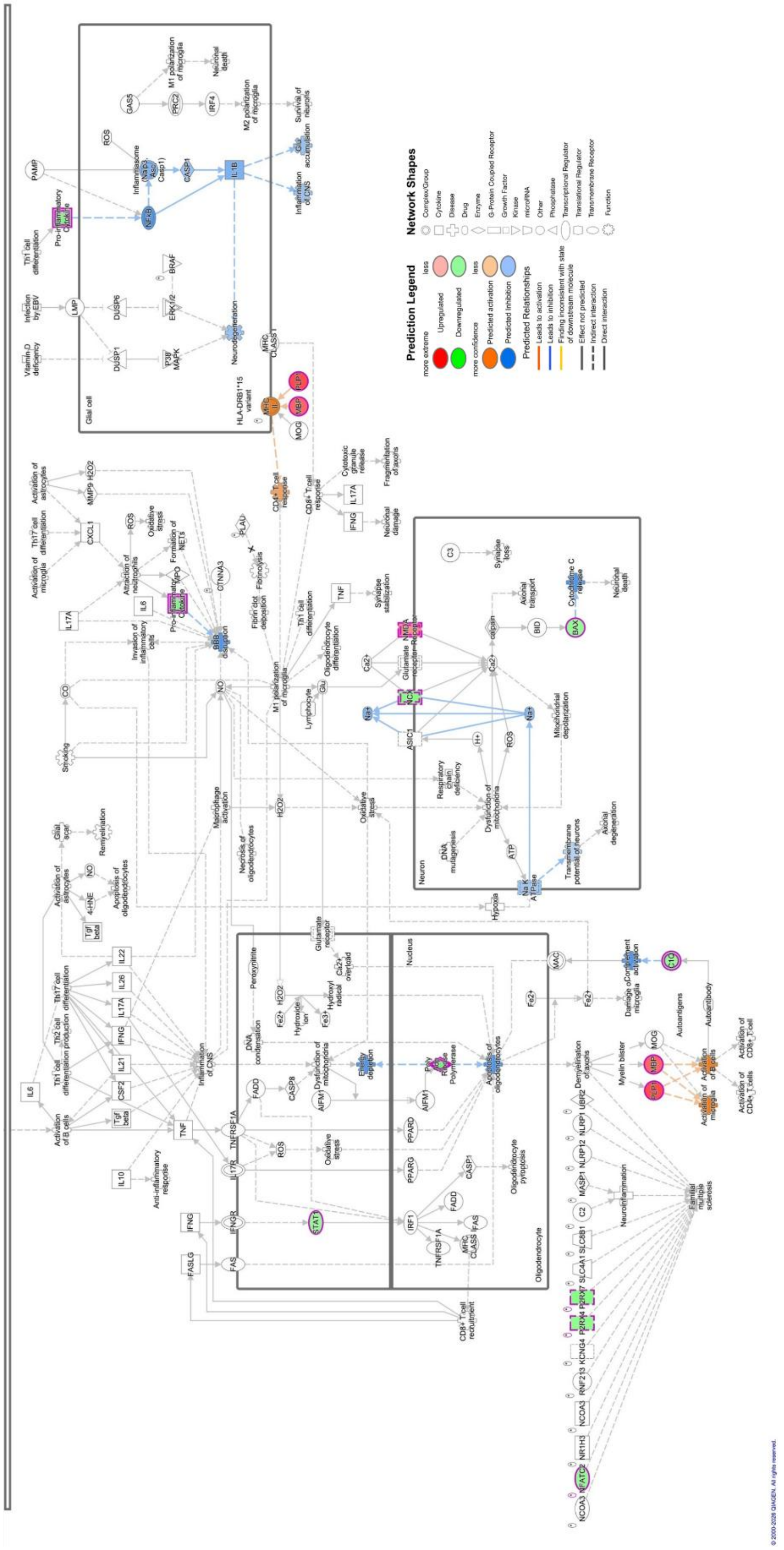

**Figure S5:** Multiple Sclerosis pathway generated from Qiagen IPA software

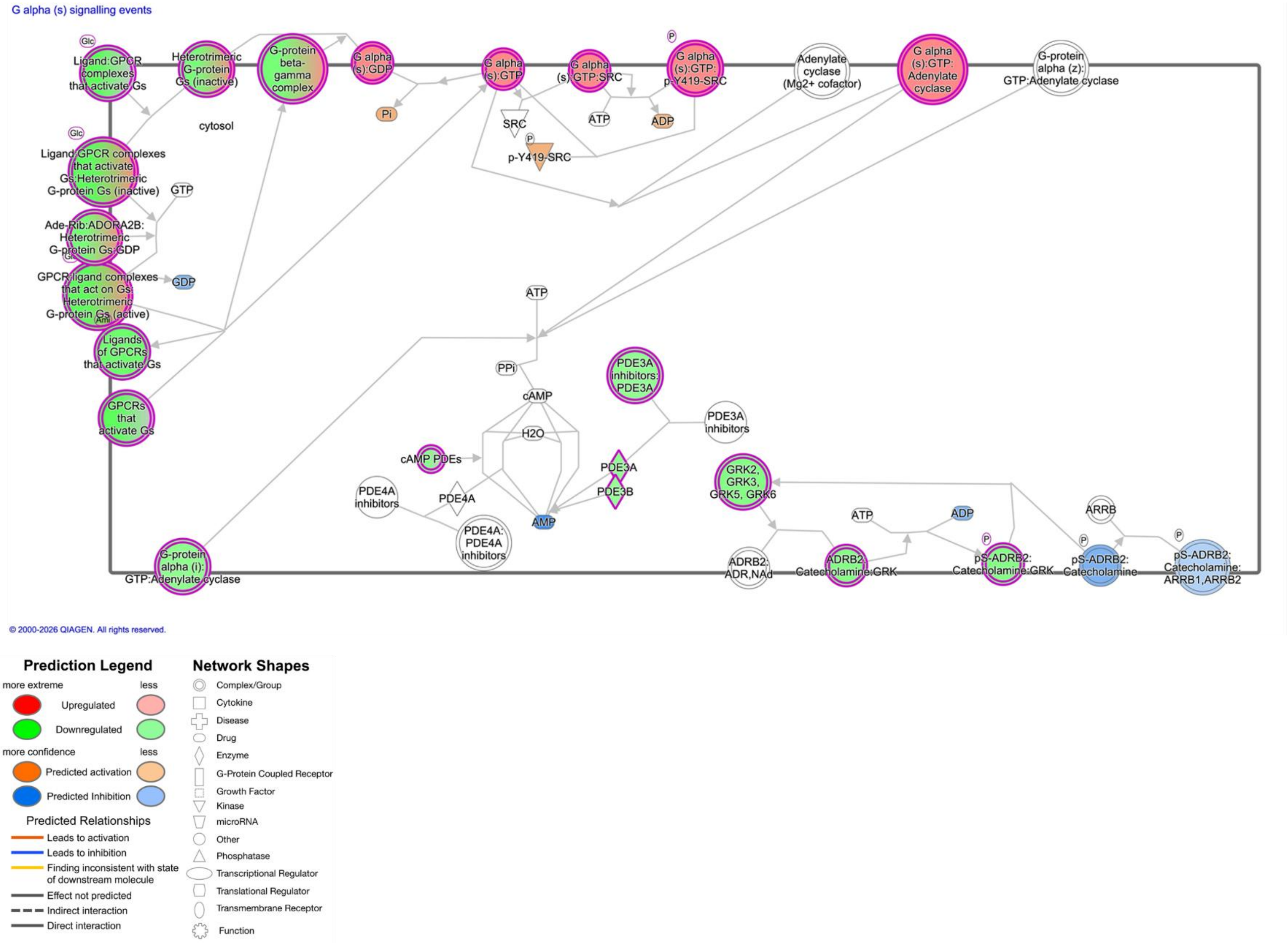


**Figure S6:** G(α)s signaling pathway generated from Qiagen IPA software.

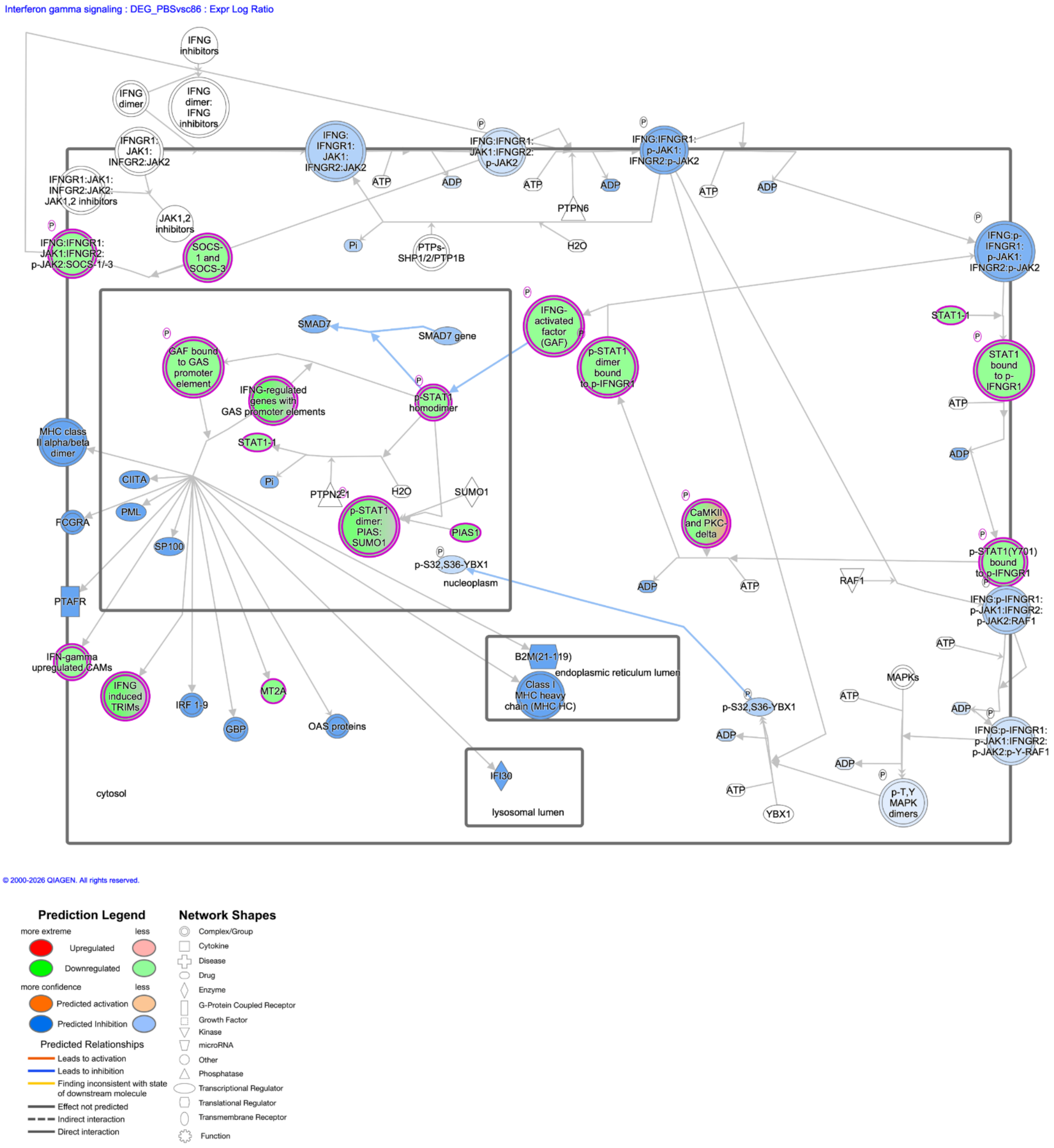


**Figure S7:** Interferon gamma signaling events pathway generated from Qiagen IPA software.

**Table S1:** qPCR primer sequences

| Primer | Forward | Reverse |
|---|---|---|
| **Mouse Gas5** | CTCCTGTGACAAGTGGAC | AACACAATATATCTGACACCATC |
| **Mouse Acot13** | GCAACCTTAGTGGACAGCATCTC | CAAGTGTCTTTCCTTGCTTCAGAA |
| **Mouse App** | TCCGTGTGATCTACGAGCGCAT | GCCAAGACATCGTCGGAGTAGT |
| **Mouse Dync1Li2** | GCTCAGACGAAGACAGTGTTGC | ATCGTGCTCCTTCTCCAAGACG |
| **Mouse Nos1** | ACCAGCACCTTTGGCAATGGAG | GAGACGCTGTTGAATCGGACCT |
| **Mouse Scoc** | AGTTGGCGTTCGAGGGCTGAAA | CGAGTCTTTTCTTCCAGTTCCAC |
| **Mouse TLR1** | GGTAGCAAGAGAAGTGGTGGAG | CGATGGTGACAGTCAGCAGAAC |
| **Mouse TLR4** | AGCTTCTCCAATTTTTCAGAACTTC | TGAGAGGTGGTGTAAGCCATGC |
| **Mouse TLR7** | GTGATGCTGTGTGGTTTGTCTGG | CCTTTGTGTGCTCCTGGACCTA |
| **Mouse TLR9** | GCTGTCAATGGCTCTCAGTTCC | CCTGCAACTGTGGTAGCTCACT |
| **Mouse Tpi1** | GGCAACTGGAAGATGAACGGGA | CTGGCAAAGTCGATGTAAGCGG |
| **Mouse Ube2n** | CCAATGGCAGCACCTAAAGTACG | GGATTGATAGCAGAACTGTGCGG |
| **Mouse Vamp2** | GAGCTGGATGACCGTGCAGATG | ATGGCGCAGATCACTCCCAAGA |
| **Mouse β-Actin** | TGTCCACCTTCCAGCAGATGT | AGCTCAGTAACAGTCCGCCTAGA |

**Table S2:** Antibodies for RNA-FISH

| Antibody | Source | Cat# | Dilution |
|---|---|---|---|
| phospho-tau s214 rabbit mAb | Cell Signaling | 77348S | 1:1000 |
| Total tau-5 mouse mAb | MilliporeSigma | MAB361 | 1:500 |
| Alexa Fluor 647 anti-rabbit | Invitrogen | A-21244 | 1:500 |
| Alexa Fluor 546 anti-mouse | Invitrogen | A-11030 | 1:500 |
| Iba1 goat mAb | Abcam | Ab5076 | 1:100 |
| GFAP rabbit mAb | MilliporeSigma | AB5804 | 1:50 |
| NeuN mouse mAb | MilliporeSigma | MAB377 | 1:50 |
| Alexa Fluor 647 donkey anti-goat | Invitrogen | A-21447 | 1:500 |
| Alexa Fluor 594 goat anti-rabbit | Invitrogen | A-11012 | 1:500 |

**Table S3:** Antibodies for Jess Automated Western Blot

| Antibody | Source | Cat # |
|---|---|---|
| pTau S214 | Cell Signaling Technology (CST) | 77348S |
| pTau202 | CST | 39357S |
| pTau217 | CST | 51625S |
| Tau-5 | Millipore Sigma | MAB361 |
| pAKT | CST | 4060S |
| tAKT | CST | 2920S |
| pGS3Kb | CST | 9336S |
| tGS3Kb | CST | 12456S |
| GAPDH | CST | 5174S |

**Table S4:** DEGs of top ssGSEA pathways

| Pathway | Estimate | P | FDR | DEGs |
|---|---|---|---|---|
| **Heme degradation** | 1.61 | 0.00012 | 0.00089 | Blvra; Hmox2; Abcc1; Slco2b1; Ugt1a2 |
| **Metabolism of porphyrins** | 2.1 | 0.000015 | 0.00089 | Blvra; Hmox2; Cpox; Abcc1; Fech; Alad; Urod; Slco2b1; Uros; Alas1; Cox15; Ppox; Flvcr1; Ugt1a2 |
| **RAF independent MAK1 3 activation** | 1.09 | 0.000035 | 0.0014 | Map2k1; Pea15a; Il6st; Dusp1; Jak2; Dusp5; Map2k2; Dusp8; Ptpn11; Dusp7; Mapk3; Mapk1 |
| **NGF stimulated transcription** | 1.14 | 0.00004 | 0.0014 | Srf; Sgk1; Nab2; Creb1; Chd4 |
| **GAB1 signalosome** | 1.11 | 0.000043 | 0.0014 | Hbegf; Pag1; Src; Pik3ca; Pxn; Tgfa; Gab1; Csk; Pik3r1; Ptpn11; Grb2 |
| **Signal regulatory protein family interactions** | -1.26 | 7.1e-7 | 0.00046 | Tyrobp; Sirpa; Cd47; Skap2; Ptk2b; Grb2 |
| **Glutamate neurotransmitter release cycle** | -0.6 | 9.0e-7 | 0.0046 | Ppfia3; Slc1a2; Slc1a3; Stx1a; Vamp2; Slc38a2; Slc1a1; Gls; Ppfia4; Stxbp1; Snap25; Unc13b; Rab3a; Cplx1; Tspoap1; Arl6ip5; Syt1; Ppfia1; Rims1; Ppfia2; Slc17a7 |
| **Protein-protein interactions at synapses** | -0.93 | 1.5e-6 | 0.0005 | Dlg3; Gria3; Grin2d; Dlgap1; Ppfia3; Homer1; Ptprs; Ppfibp1; Grm1; Epb41l2; Gria1; Grin2c; Dlg4; Il1rap; Shank3; Dlg1; Epb41l3; Nrxn1; Rtn3; Homer2; Gria4; Ppfia4; Sh3glb2; Grin1; Ptprd; Epb41; Lrfn1; Cask; |

| | | | | |
|---|---|---|---|---|
| | | | | Nlgn3; Slitrk5; Ptprf; Nrxn2; Ppfibp2; Slitrk2; Lrfn3; Ppfia1; Shank2; Shank1; Lrfn2; Dlgap3; Lrrtm3; Lrfn4; Slitrk6; Slitrk4; Lrrc4b; Dlgap2; Slitrk3; Grm5; Nlgn2; Il1rapl1; Dlg2; Lrrtm4; Ppfia2; Grin2a; Ntrk3; Flot1; Lrrtm1; Dlgap4; Flot2; Nlgn1; Nrxn3; Lrrtm2; Slitrk1 |
| **Receptor type tyrosine protein phosphatases** | -2.01 | 0.000002 | 0.0005 | Ppfia3; Ptprs; Ppfibp1; Il1rap; Ppfia4; Ptprd; Slitrk5; Ptprf; Ppfibp2; Slitrk2; Ppfia1; Slitrk6; Slitrk4; Lrrc4b; Slitrk3; Il1rapl1; Ppfia2; Ntrk3; Slitrk1 |
| **Sumoylation of transcription factors** | -1.65 | 2.8e-6 | 0.0005 | Pias4; Ube2i; Sumo3; Sumo2; Mta1; Pias2; Sumo1; Sp3; Pias3; Pias1; Trp53bp1 |

**Table S5:** DEGs of top ORA pathways

| ORA PathwaysIngenuity Canonical Pathways | -log(p-value) | Ratio | z-score | Molecules |
|---|---|---|---|---|
| **Multiple Sclerosis Signaling Pathway** | 3.42 | 0.119 | -3.922 | BAX, C1QA, GRIN1, MBP, NFATC2, P2RX4, P2RX7, PARP10, PARP12, PARP14, PARP2, PARP9, PLP1, SLC8A3, STAT1, TLR1, Tlr11, TLR4, TLR5, TLR6, TLR8, TLR9, TNFSF14, TNFSF4, TNFSF8, TNFSF9 |
| **Parkinson's Signaling Pathway** | 6.49 | 0.134 | -3.795 | ATP6V0A1, ATP6V1D, ATP6V1E1, CACNA1B, CACNB4, CACNG5, CALM2, CAMK2A, CAMK2D, CASP9, GRIA4, GRIN1, LRRK2, NDUFA3, NDUFA6, NDUFB1, NDUFB2, NDUFB8, NDUFS2, NDUFS5, NDUFS6, NOS1, PINK1, SLC18A2, SLC6A3, TH, TLR1, Tlr11, TLR4, TLR5, TLR6, TLR8, TLR9, TNFSF14, TNFSF4, TNFSF8, TNFSF9, TOMM20, TUBA1B, TUBB1, UCHL1, ZNF746 |
| **G alpha (s) signalling events** | 4.06 | 0.145 | -3.71 | ADCYAP1, GNAI1, GNAS, GNG10, GNG3, GNG7, GRK3, HTR6, HTR7, PDE10A, PDE11A, PDE1B, PDE3A, PDE3B, RAMP2, RAMP3, |

| | | | | |
|---|---|---|---|---|
| | | | | RXFP2, TAAR1, TAAR5, TAAR6, TSHR |
| **Interferon gamma signaling** | 2.4 | 0.133 | -3.051 | CAMK2A, CAMK2D, MT2A, PIAS1, SOCS3, STAT1, TRIM10, TRIM14, TRIM21, TRIM25, TRIM29, TRIM31, VCAM1 |
| **Neuroinflammation Signaling Pathway** | 2.55 | 0.097 | -3.024 | APP, CALB1, CALB2, CX3CL1, ELP1, GABRA1, GABRA2, GAD1, GRIN1, IKBKB, MFGE8, NFATC2, P2RX7, PLA2G4F, PLA2G6, PPP3CB, PPP3R1, PSEN2, REL, S100G, STAT1, TICAM2, TLR1, Tlr11, TLR4, TLR5, TLR6, TLR8, TLR9, TREM2, VCAM1, ZBTB12 |

**Data S1:** (separate file) Raw DSP data.
